# How the Nobel Committee for Chemistry Has Shaped the Nobel Prize: Historical Trends Based on the Nobel Prize Nomination Archive

**DOI:** 10.1021/acsomega.4c08461

**Published:** 2025-05-13

**Authors:** Jeffrey I. Seeman, Juan Amaya, Guillermo Restrepo

**Affiliations:** † Department of Chemistry, University of Richmond, Richmond, Virginia 23173, United States; ‡ Technische Universität Dresden, 01062 Dresden, Germany; § Max Planck Institute for Mathematics in the Sciences, Inselstraße 22, 04103 Leipzig, Germany; ∥ Interdisciplinary Center for Bioinformatics, Leipzig University, Härtelstraße 16-18, 04107 Leipzig, Germany; ⊥ School of Applied Sciences and Engineering, EAFIT University, 050022 Medellín, Colombia

## Abstract

The Nobel Prize in Chemistry is governed by the Nobel Foundation’s rules and managed by the Royal Swedish Academy of Sciences via its Nobel Committee for Chemistry. Despite 125 years of awarding these prizes and increased transparency from the Nobel Foundation, many aspects of the selection process remain obscure. Our analysis of the Nobel Prize Nomination Archive from 1901 to 1970 reveals that the decisions were heavily influenced by the personal and professional biases of the members of the Nobel Committee for Chemistry, who were not only responsible for inviting and assessing nominations but also for presenting recommendations to the Academy. Nobel laureates also significantly influenced the process due to their right to make ongoing nominations. The study suggests ways to improve the nomination and selection process to better reflect the evolving nature of chemistry in the 21st century.

## Graphical abstract

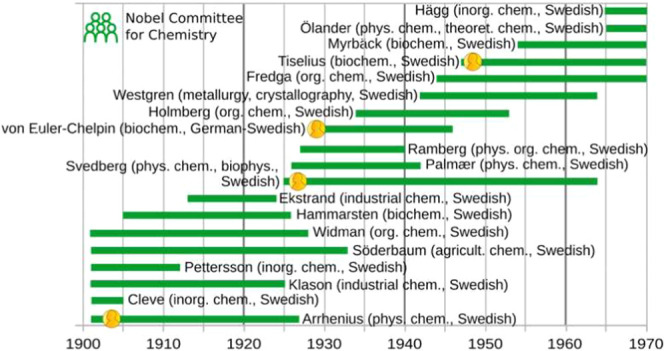

## 1 Introduction

The Nobel Prize in Chemistry (NPch) is widely regarded as the highest honor in the discipline.^1–9^ The NPch celebrates remarkable contributions to chemistry, carries significant global influence, and boasts a legacy of over a century. NPch recipients are expected to have had a profound impact on the chemical community and society, at large. Receiving this prestigious award often opens doors to greater funding, resources, and opportunities, which can further propel research and contribute to advancements in science and technology worldwide. Therefore, studying and understanding the driving forces shaping the NPch is an opportunity to understand and even enhance productivity in chemistry.

Using electronic records of the Nobel Prize nomination process from 1901 to 1970 for the NPch, we conducted a study aimed at uncovering insights into the inner workings of the recipient selection process for the NPch. Our findings, combined with historiographical research on the NPch, offer a detailed and nuanced view of the nomination and selection procedures. Similar studies on the Nobel Prize in Physics^10,11^ and the Nobel Prize in Physiology or Medicine^12–14^ were recently published, alongside other data-driven analyses of the Nobel Prize system.^11,15–26^

Our data-driven approach asks: What has been the role of the number of nominations and of the number and types of nominators upon the selections of the NPch? Does the participation of nominators follow a homogeneous or rather a biased trend? How successful are nominators in their nominations, and what is the nature of successful nominators? Can Nobelists be quantitatively differentiated from non-Nobelists based on proxies such as the number of nominations received and the time spanned by those nominations?

In order to set the stage for our study, we describe in the next section the nomination process.

### 1.1 Nomination Process

Between September and October of each year, the Nobel Committee for Chemistry (the “Committee”), selected by the Royal Swedish Academy of Sciences, solicits nominations for the following year’s NPch. According to the rules developed by the Nobel Foundation, nominations can only be submitted by “1. Swedish and foreign members of the Royal Swedish Academy of Sciences; 2. Members of the Nobel Committees for Chemistry and Physics; 3. Nobel Prize laureates in chemistry and physics; 4. Permanent professors in the sciences of Chemistry at the universities and institutes of technology of Sweden, Denmark, Finland, Iceland, and Norway, and Karolinska Institutet, Stockholm; 5. Holders of corresponding chairs in at least six universities or university colleges selected by the Academy of Sciences with a view to ensuring the appropriate distribution over the different countries and their centers of learning; and 6. Other scientists from whom the Academy may see fit to invite proposals.”^27^

January 31st of each year is the deadline for receipt of nominations. Nominations are then reviewed and assessed by the Committee. The following September, the Committee submits their recommendations to the Academy. In early October, the Academy selects the NPch laureates through a majority vote of its members. The decision is irrevocable, and shortly thereafter, the names of that year’s Nobelists are announced to the recipients, then to the general public. Early in December, the Nobel Prize Award ceremony takes place in Stockholm.^27^

In the next section, we provide details on the data used to carry out ourstudy.

### 1.2 Data and Definitions

The data was retrieved from the Internet site of the Nobel Foundation^28^ and is available in the Supporting Information (SI 1–1). All analyses were conducted using data classified under the NPch category. According to the Nobel Foundation’s regulations, information about nominations cannot be disclosed publicly or privately for 50 years. The data set, retrieved in September 2022, spans the years 1901–1970 and remains the only period available as of April 2025. Our data set gathers 4,325 nominations involving 1610 nominators and 646 nominees (see SI 1–1 and SI 1–2).

We analyzed the entire period 1901–1970 as a whole as well as the following three time-periods: 1901–1920, 1921–1940, and 1941–1960. We did not analyze the decade 1960–1970 as a specific group, as most of the effects of the nominations for these years will be evident only *after* further years of data are released by the Nobel Foundation.

We have defined and used certain terms that are useful in presenting and discussing the results in this publication.

We define a “*nomination commitment*” or more simply a “*commitment*” as the event that represents a directed nominator → nominee relationship. Whenever a nominator *A* nominates candidate *B*, the nomination produces a commitment of *A* to *B* (or *A* → *B*). When *A* commits to *B*, *A* can express their commitment through one or more nominations, depending on how often *A* is allowed to nominate and wishes to nominate *B*.

We define several types of nominators:

- *Permanent nominators* have the right to make a nomination every year. They include ex-officio nominators, who are members of the Royal Swedish Academy of Sciences, members of the Nobel Committees for Chemistry and Physics, Nobel Prize laureates in chemistry and physics, permanent professors in the science of chemistry at the universities and institutes of technology of Scandinavian countries and Karolinska Institutet.
- *Ad-hoc nominators* are holders of corresponding chairs in at least six universities or university colleges selected by the Academy of Sciences and other scientists from whom the Academy may see fit to invite nominations.
- *Regular nominators* nominate once or infrequently.
- *Frequent nominators* often submit nominations.
- *One-shot nominators* make just one nomination and therefore commit to a single candidate during the time period of the data set (1901–1970).
- *Monogamous nominators* commit to a single nominee through several nominations.
- *Polygamous nominators* commit to several nominees.
- *Successful nominators* are those for whom more than half of their nominees receive the NPch.
  Specific kinds of nominations are
- *Aligned nominations* are made by several nominators for the same nominee.
- *Superabundance of nominations* quantifies the number of nominations greater than one that a nominator makes per commitment.
- *Nomination trajectories* are temporal curves of the number of nominations a nominee receives.

## 2 Results and Discussion

### 2.1 A Growing Enterprise: Number of Nominators, Nominees, and their Associated Commitments and Nominations

A rather stable trend of the number of commitments and nominations was found for the first 45 years of the award (1900s through the mid-1940s), with about 28 commitments and 37 nominations in each year (Figure 1a) (that there are less commitments than nominations reflects multiple nominations by the same nominator for the same nominatee during the stated time period). After World War II (WWII) the rate of nominations rapidly increased, with the exception of a strong drop in 1970 for which we cannot propose any reason. This post-WWII trend for nominations was noticed previously.^29^ The number of commitments and nominations is strongly correlated (0.9872, *p*-value <2.2 × 10^–16^) (Figure 1a), indicating how intertwined nomination and commitment dynamics have been over the first 70 years of the NPch.

**Figure 1:**
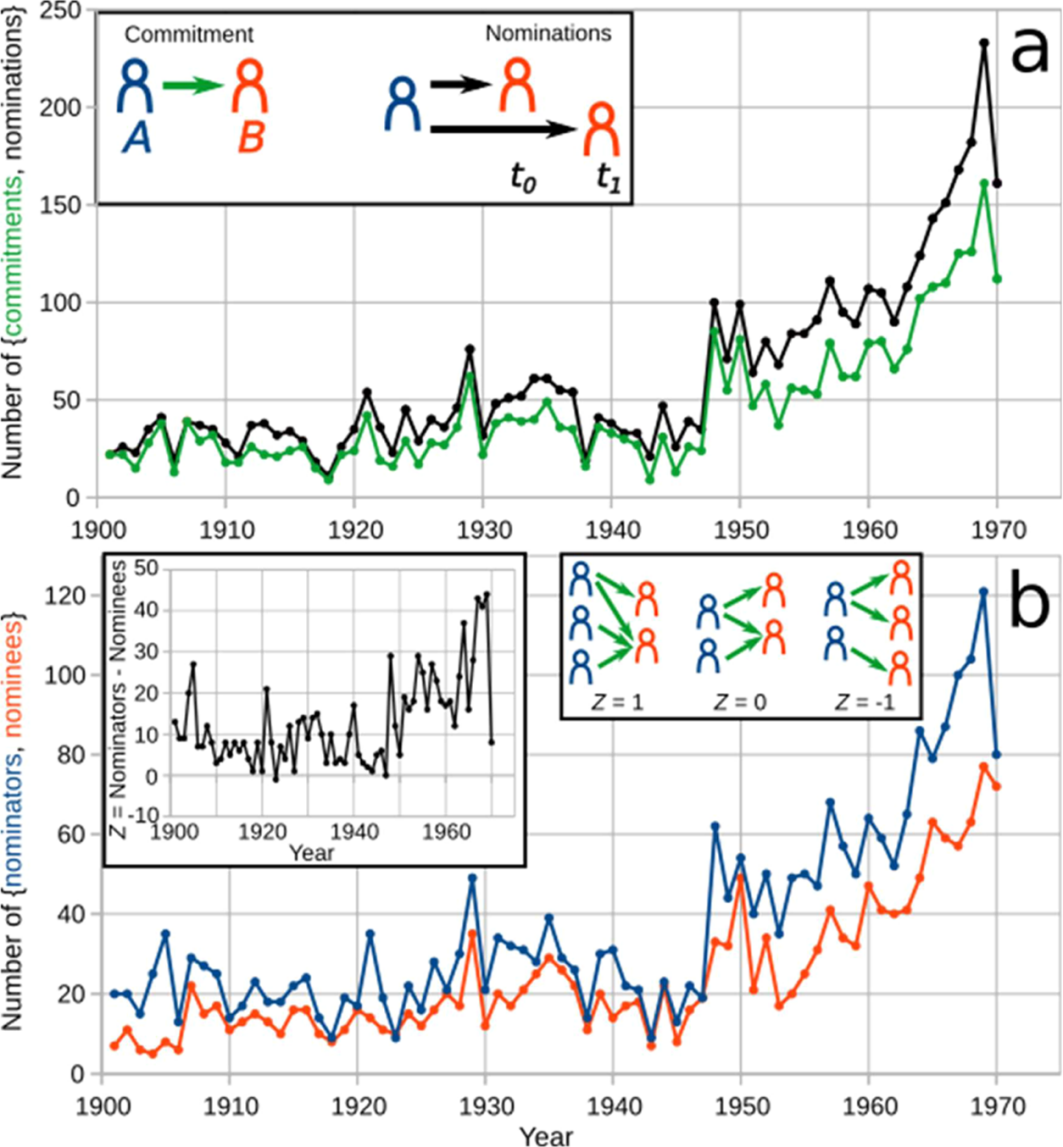
Nomination pool. Number of (a) commitments and nominations for the Nobel Prize in Chemistry initiated every year and the associated number of (b) nominators and nominees over the time period 1901–1970. Inset in (a): A commitment represents the endorsement of nominator *A* for nominee *B* as a candidate for the Nobel Prize in Chemistry. Every commitment involves at least one nomination, represented as two nominations of *A* for *B*, one in year *t*_0_ and the other in year *t*_1_. Left inset in (b): Difference *Z* between the number of nominators and of nominees for each year. Right inset in (b): Schematic representation of three cases with different numbers of nominators (blue), nominees (orange), and commitments and nominations (green arrows) with their respective *Z* values. Note that in *Z* = 1, there are three aligned nominations, while in *Z* = 0 two and in *Z* = −1 none.

The number of nominators and of nominees is also correlated (0.9408, *p*-value <2.2 × 10^–6^), (Figure 1b). This implies that the size of the community of nominators is proportional to the size of the community of nominees and the converse. Figure 1b also shows that the historical trend has been to have more nominators than nominees, which indicates that over time there has always been a certain degree of *aligned nominations*, that is, a matching of several nominators’ candidates (Figure 1b, insets). Otherwise, if every nominator had nominated candidates who were not nominated by any other nominator, the number of nominees would be equal or greater than that of nominators. Although not the historical trend, there have been such alternative scenarios (Figure 1b, left inset) in World War periods. This oddity suggests that during WWs, reaching consensus among nominators about possible Nobelists becomes difficult for the Committee. Further discussion on the effects of WWs is provided below.

Figure 1b indicates that the stability of pre-WWII times in terms of nominations and commitments (Figure 1a) went hand-in-hand with a steady number of nominators and of nominees, with about 24 nominators nominating about 15 nominees. The surge of commitments and nominations after WWII (Figure 1a) coincides with a corresponding growth in the number of nominators and nominees (Figure 1b). The chemical community has grown exponentially at least from the 19th century up to date.^30,31^

Overall, Figure 1 indicates that the NPch grew steadily in the number of commitments, nominations, nominators, and nominees throughout the first four decades of the award. Nomination activity declined during both WWs, with the NPch being temporarily halted during those periods. After WWII, the NPch experienced renewed vigor, marked by a sharp rise in nominators, nominees, nominations, and commitments. Historically, there have consistently been more nominators than nominees, indicating a notable level of alignment in nominations, where nominators often chose the same candidates.

Only after WWII has the number of nominators reflected the growth of the chemical community. Was the surge of nominators after WWII the result of a counterreaction to extreme nationalistic ideas of pre-WWII times? Besides the reasons triggering this post-WWII surge in participation in the nomination process, is the post-WWII NPch different from that of previous years? That is, is there a different distribution of the number of commitments and nominations over the population of nominators and nominees before and after WWII? Did more nominators and nominees set a new stage for the NPch? How did the Nobel committee handle the voices of a growing number of nominators? We have analyzed these questions in the next several sections.

We next divide our analysis into two primary domains: one focusing on the nominators and their involvement in the nomination process and another concentrating on the role of the nominees.

### 2.2 Nominators

#### 2.2.1 Nominators Allowed by the Statutes and the Two Extreme Scenarios for their Participation in the Nomination Process

As a consequence of the bylaws of the Nobel Prize, only a small and select group of individuals, mainly Scandinavians and non-Scandinavians chosen by Scandinavians, have the right to nominate. Of those nominators, some *permanent nominators* are eligible on an annual basis while others, the *ad-hoc nominators*, are invited on an irregular basis.^5^ The former are Scandinavians—including Committee members and members of the Royal Swedish Academy of Sciences—plus Nobelists, while ad-hoc nominators include primarily non-Scandinavian academics of high ranking, that is, at the professorial level.

The distinction between permanent and ad-hoc nominators leads to two extreme scenarios in terms of the degree of participation of nominators. This participation is reflected in the number of nominations submitted by every nominator. The two scenarios are:

(1) *Even participation of nominators*: When permanent and ad-hoc nominators nominate with similar frequency, therefore, no clear distinction among nominators in terms of their number of commitments and nominations is observed. This corresponds to a homogeneous distribution of the number of commitments and nominations over the population of nominators. In this scenario, no nominator had more chances to push for their candidates.
(2) *Uneven participation of nominators*: Some nominators nominate more than others, distinguishing themselves in terms of their number of commitments and nominations. A heterogeneous distribution of the number of commitments and nominations over the population of nominators occurs. Hence, some nominators have more chances to advance their candidates.

We next explore the participation of nominators and their effects upon the NPch.

#### 2.2.2 Regular and Frequent Nominators

To quantify the participation of nominators in the nomination process, we analyzed how frequently nominators were committed to nominees by their frequency of submitting nominations. We determined the percentage of nominators who contributed within a specified number of commitments/nominations (Figure 2).

**Figure 2:**
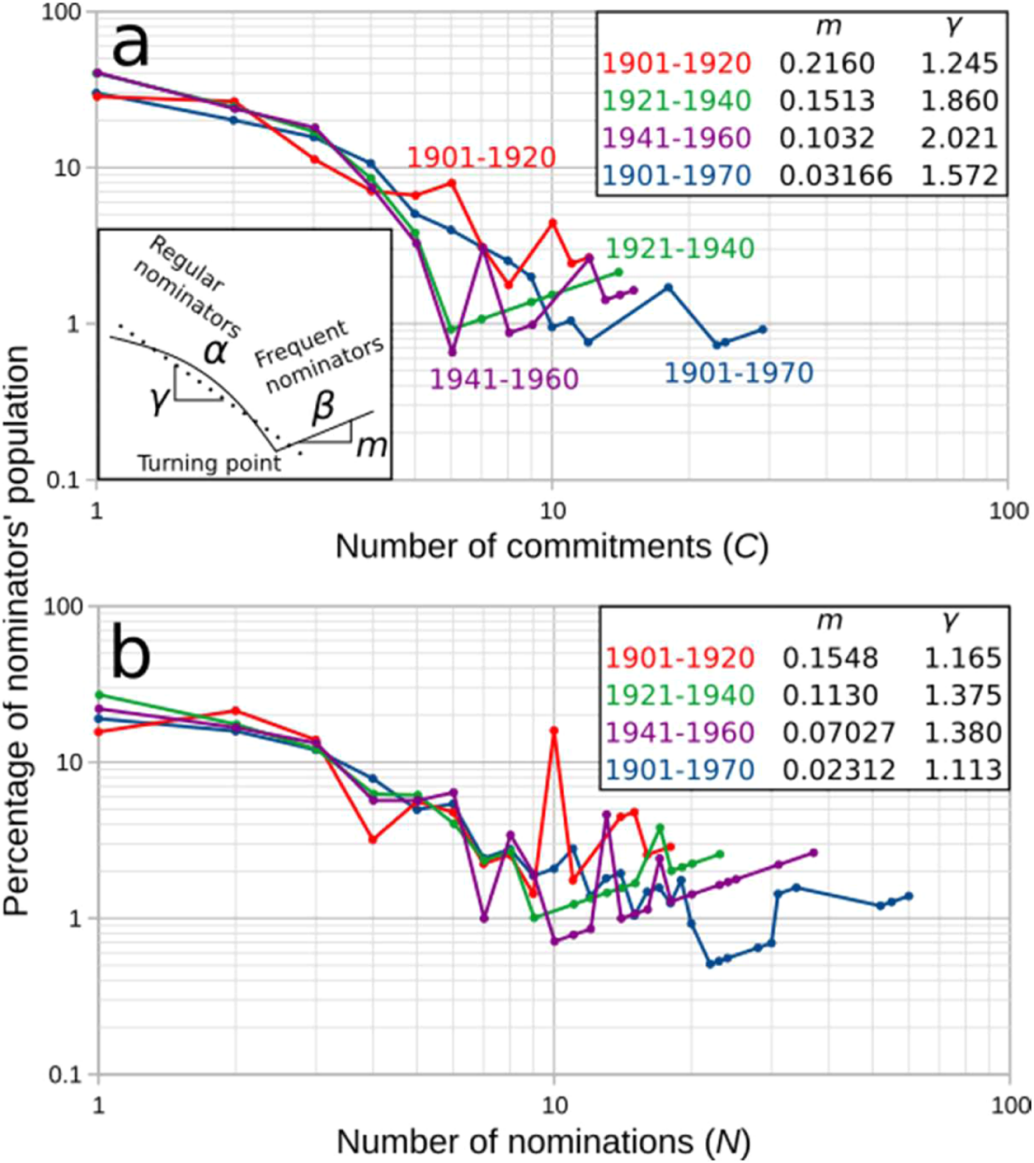
Nominators’ participation. Percentage of nominators (a) committed to *C* nominees and (b) submitting *N* nominations. Inset in (a) - (bottom) models the distributions shown in (a) and (b) and divides those distributions into α and β regions, which characterize regular and frequent nominators, respectively. These regions are separated, in each period, by a turning point, where the percentage of the nominators’ population is the lowest. α region is roughly approximated with *P*_α_(*X*) ∼ *X* ^–γ^, with *P*_α_(*X*) indicating the percentage of nominators on the α region and *X* being either *C* or *N*. γ corresponds to the slope of the fitting curve in the α region. The higher γ, the less homogeneous the participation of regular nominators is. β region depicts a trend of the form *P*_β_(*X*) ∼ *X* ^*m*^ (or equivalently *log*(*P*_β_(*X*)) ∼ *m*log­(*X*)), with *m* being the slope of the fitting curve (after removing outliers and running a least-squares fiiting, see SI 1.3). The higher *m*, the less homogeneous the participation of frequent nominators is. Tables in (a) and (b) correspond to the values of γ and *m* for the analyzed periods.

Over the period studied, many nominators committed to one or several nominees through a few nominations, while a small fraction of nominators committed to many nominees (Figure 2a,b). This is evident in the two kinds of behavior (regions α and β in the inset of Figure 2a), which separate nominators into *regular* and *frequent nominators*. The vast majority of nominators are regular nominators, characterized by a wide range of commitments/nominations. These nominators span the region α (note that over history, about 80% of the nominators lie in the region α (Figure 2a,b)). These nominators include *one-shot nominators*, who commit to a single nominee through a single nomination, at the left-hand side of region α in Figure 2a,b as well as nominators with more than one commitment/nomination, who populate the region α until reaching its lowest value at the juncture with region β (turning point in Figure 2a,b).

The population (*P*_α_) of regular nominators can be approximated to a power-law fitting curve *P*_α_(*X*) ∼ *X* ^–γ^,^32^ with *X* representing either the number of commitments or of nominations of the population of regular nominators. In this power-law equation, γ measures the increase in heterogeneity in the number of commitments and nominations submitted by regular nominators. As shown in Figure 2a,b (tables), γ has increased over time. Therefore, the passage of time has led to a more heterogeneous participation of regular nominators in the NPch nomination process. A mechanism leading to power-law distributions, as those observed in regions α of Figure 2a,b, and often found in social systems,^32^ is based on a preferential attachment model.^33^ This model implies that regular nominators who have committed to several nominees are rare, but over time, they tend to increase their number of commitments. In contrast, regular nominators with a few commitments tend to remain with few commitments.

Based on the lowest γ values of the first analyzed period, the increase of γ (Figure 2a,b) indicates that the first two decades of the 20th century were the times of least heterogeneous participation of regular nominators. This provides quantitative support to the historiographical consideration that at the turn of the 20th century, there was a strong internationalistic spirit.^5,34^ The subsequent increase of γ indicates a more heterogeneous participation of regular nominators. That is, nominations and commitments began to concentrate on some nominators, which is presumably caused by a preferential attachment process as explained in the previous paragraph.

Figure 2 illustrates that between the two scenarios outlined in Section 2.2.1—namely, equal versus unequal participation of nominators in the NPch nomination process—the latter scenario has occurred. Over the analyzed history of the NPch, the nominator population distinctly falls into two categories: a large majority of regular nominators and a smaller group of frequent nominators. We asked: What are the identities of these frequent nominators? What is the cause of the emergence of frequent nominators? The first question is the subject of Section 2.2.3 and the second is addressed in Section 2.2.4.

#### 2.2.3 Frequent Nominators Correspond to a Few Committee Members and Nobelists

To determine the identities of frequent nominators, we plotted the number of commitments and nominations for each nominator (1901–1970) (Figure 3). In Figure 3, the turning points of Figure 2 that separate regular from frequent nominators are indicated with vertical dotted lines. Frequent nominators are located at the right-hand side of the vertical dotted lines in each plot. Just a few Committee members and Nobelists correspond to frequent nominators, while regular nominators span the rest of the nominators, these being at the left of the vertical dotted lines (Figure 3). Figure 3 also shows an important historical trend: a matching of frequent nominators with a reduced set of Committee members and Nobelists. These frequent nominators who were Committee members include Hans K. von Euler-Chelpin and Theodor Svedberg (the most frequent nominators ever), along with Nobelist Harold C. Urey (Figure 3a). In Figure 3b it is observed that the most frequent nominators in the first two decades of the award were Nobelists Adolf von Baeyer, Emil H. Fischer, and Svante A. Arrhenius, the latter also a Committee member of the Physics Prize with strong influence in the NPch.^5^ Figure 3c depicts the interwar period, where Svedberg and von Euler-Chelpin became the most frequent nominators as well as the Nobelists Walther H. Nernst, Richard M. Willstätter, and Max K. Planck. The post-WWII decades show the leading role of Committee member von Euler-Chelpin as frequent nominator, accompanied by Committee members Svedberg and Arne Tiselius and by the Nobelists Otto Hahn, Leopold Ružička, Urey, John H. Northtrop, and Adolf F. Butenandt. Figure 3 shows that frequent nominators correspond to a particular subset of permanent nominators (Section 2.2.1) who were Committee members and/or Nobel laureates.

**Figure 3:**
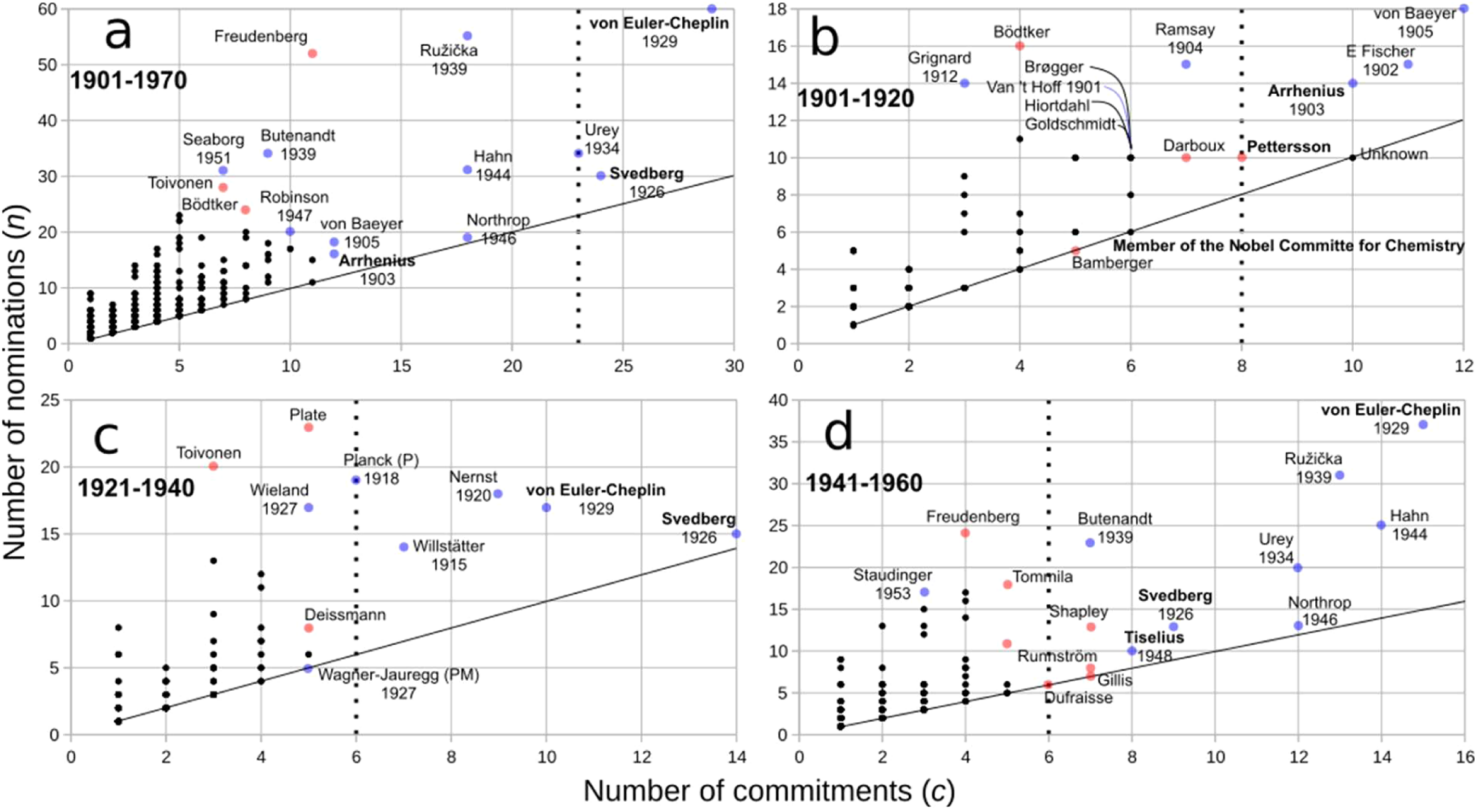
Nominators’ pressure for their candidates. Scatter plot of number of nominations (*n*) and number of commitments (*c*) per nominator during the indicated periods. Colored dots correspond to nominators with relatively high *n*. Red dots indicate nominators who did not receive the Nobel Prize in the period analyzed and blue dots indicate those who did. For the latter, the awarding year is provided. Note that, especially at low *n* and *c* values, every dot may correspond to more than one nominator (black dots), where we do not differentiate Nobelists from non-Nobelists. Vertical dotted lines separate regular from frequent nominators (Figure 2); the latter are located to the right of the lines. Values of *c* to draw these lines were selected from the turning point values of *C* in each period analyzed in Figure 2, where regions α and β meet. Nominators in boldface correspond to members of the Nobel Committee for Chemistry. Diagonal black lines indicate *n* = *c*, that is, a theoretical line representing cases in which each commitment is met with only one nomination. Note that as *n* ≥ *c*, no dots are possible below the diagonal line. The distance above this line represents the *superabundance of nominations* nominator *A* has for any one nominee, which is calculated as *S*(*A*) = *n*(*A*) – *c*(*A*), with *n*(*A*) and *c*(*A*) being the nominations and commitments of nominator *A*, respectively (see Section 2.2.5 for a discussion on superabundance). In (b) “Unknown” corresponds to a nominator with no name nor internal identification number in the Nomination Archive. In (c), “P” and “PM” stand for Nobel Laureates in Physics and in Physiology or Medicine, respectively.

#### 2.2.4 Statutes Contribute to the Emergence of Committee Members and Nobelists as Frequent Nominators

In Section 2.2.3, we found that some of the frequent nominators are Committee members and Nobelists. These kinds of nominators are two representatives of the permanent nominators’ pool, who accompany the members of the Academy and the group of Scandinavian chemistry professors (Sections 1.2 and 2.2.1). Why did only Committee members and Nobelists and not other permanent nominators become frequent nominators? Or more generally, why did other nominators such as ad-hoc nominators not become frequent nominators? (Section 1.2).

We hypothesize that nominators who have more time as permanent nominators became frequent nominators. To test this hypothesis, we calculated the time of service as nominators of permanent nominators and also of ad-hoc nominators. The time of service is of two sorts: potential and actual. The *potential* time of service of a nominator is calculated as the time the nominator holds a position allowing them to nominate, that is, as a member of the Academy, the Committee, or as a Nobelist. The *actual* time of service of a nominator corresponds to the time spanned between the nominator’s first and last nominations.

The average potential time of service as nominators of Academy members was 25.4 ± 2.0 years (for details see SI 1–4). The actual time as nominators could not be calculated, as only six out of the 55 Academicians belonged to the sample who ever submitted nominations. This result indicates that even when Academicians had more than two decades to nominate, most seldom, if ever, made nominations. The lack of participation by the Academicians, in contrast to the Committee members, all of whom are also members of the Academy, is noteworthy.

For Nobelists, the potential time span for serving as nominators ranged from the year after they received the Nobel Prize until their death, averaging 23.8 ± 1.4 years (SI 1–4). However, the actual (observed) average duration of their nominations was shorter, lasting 15.0 ± 1.4 years between their first and last nominations (SI 1–4). Nobelists do not always make nominations *after* receiving the award, but interestingly, they often appear as nominators *before* becoming Nobelists. In some instances, they serve as ad-hoc nominators, while others, particularly those affiliated with Scandinavian institutions, become permanent nominators. These findings indicate that laureates have a similar potential time frame for nominations as Academicians. However, unlike Academicians, Nobelists actively use this opportunity, focusing their efforts within a roughly 15-year window. Figure 4b highlights the significant role some Nobelists play as frequent nominators for the NPch.

**Figure 4:**
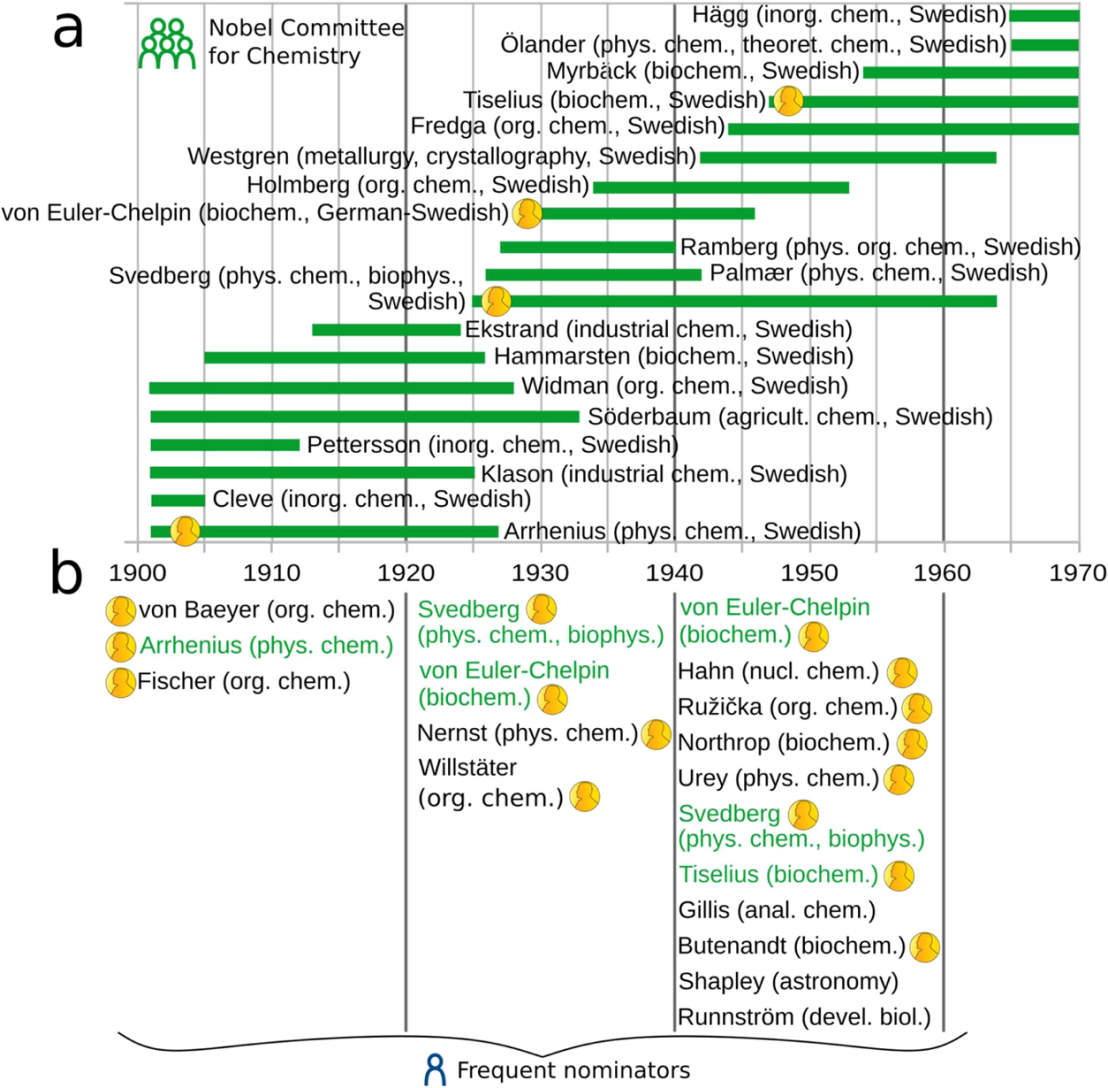
Constitution of the Committee and its interplay with frequent nominators, including Nobelists. (a) Infographic depicting the period of service of all regular members of the Nobel Committee for Chemistry between 1901 and 1970, plus Svante A. Arrhenius, a member of the Nobel Committee for Physics but highly influential in the Nobel Prize in Chemistry. In all cases, nationalities are provided in parentheses. For Committee members who became Nobelists in chemistry, a Nobel Prize medal is depicted for the awarding year. (b) Frequent nominators in different periods of the history of the Nobel Prize in chemistry (see Figure 3). Those in green correspond to Committee members, and those with the Nobel prize medal are Nobelists. For each scientist, their field of work is provided. Original graphic based on literature information.^3,5,6,16^

Other permanent nominators are Committee members, who have the greatest degree of potentially excessive influence as they can both nominate and select! (Our analysis did not consider adjoint members of the Committee.^16a^) Committee members’ potential time as nominators was calculated as the difference between the first and last year of membership to the Committee (Figure 4a). The resulting 20.2 ± 2.1 years of potential time as nominators was extended to 21.7 ± 2.9 years, which corresponds to the actual time as nominators for Committee members (SI 1–4). This extension occurs because Committee members can nominate even before becoming Committee members, given their membership to Scandinavian institutions. This latter membership turns them into permanent nominators. Interestingly, of all permanent nominators, Committee members are those who actually nominated for the longest period. In fact, they did it in the analyzed period (1901–1970) for about 22 years. The active role of some Committee members as frequent nominators to the NPch is evident in Figure 4b.

For comparative purposes, we calculated the actual time of service of ad-hoc nominators, which turned out to have a median value of 3.59 ± 0.17 years. The brevity of this period is due to a second observation, that the majority of these ad-hoc nominators nominated only once.

Remarkably, the above results reject the hypothesis that, for the entire body of nominators, those with longer times of service make more nominations. If it were true, members of the Academy would be the most frequent nominators. In contrast, other individuals who are both frequent and permanent nominators are those who have submitted the most nominations, namely, Committee members and Nobelists. Their frequent participation in the nomination process is the result of an opportunity provided by the statutes of the Nobel Prize. While Nobelists have more potential time to nominate, it is the Committee members who actually nominated more frequently. That is, from 1901 to 1970, a group of 19 Committee members with the option of nominating in an average period of about two decades had the largest presence in the nomination process. Indeed, they had a presence much larger than the actual nomination time of the 84 Nobelists, who were nominated for about 15 years. Nonetheless, the actual nomination time of Committee members (average = 22 years) is lower than the potential nomination time of Academicians (average = 25 years). The role of the Academy’s membership on the fabric of the NPch could, in principle, have been even greater had they been more motivated to nominate and had taken their opportunities to nominate. Thus, it is not the active time as nominators that drives the emergence of Committee members and Nobelists as frequent nominators.

We next turn to explore the details of the nomination process, analyzing the strength of the nominator’s individual commitments.

#### 2.2.5 Frequent Nominators Push One-at-a-Time

We considered whether frequent nominators were *polygamous nominators*, nominating several individuals in the same year, or whether they were, in contrast, *monogamous nominators*, concentrating their nominations on single candidates, one at a time. We analyzed the relationship between the number of nominations associated with the commitments of each nominator. The correlation between the number of nominations and of commitments, as observed in Figure 3a–d, is high (0.860, *p*-value <2.2 × 10^–16^). This is because most of the population of nominators is spanned by one-shot nominators, who are located close to the diagonal in Figure 3a–d. But there are nominators submitting more than one nomination per commitment. These nominators are located above the diagonal of Figure 3a–d. For every nominator, their vertical distance from the diagonal quantifies the perseverance of their commitments through nominations, as it indicates how many additional nominations to the initial single nomination per commitment the nominator submitted. We call this distance the *nominator’s superabundance of nominations*.

Figure 3 shows that there are no clear differences between frequent and regular nominators (the former on the right of the dotted lines in Figure 3a–d and the latter on the left) in terms of their superabundance of nominations. For both regular and frequent nominators, there are individuals who, instead of venturing into new commitments, preferred to push for their already committed nominees.

But how did superabundant nominators distribute their nominations? Did nominators with superabundant nominations push homogeneously for their nominees and distribute nominations proportionally over their candidates, turning these nominators into polygamous frequent nominators, or alternatively, did they concentrate their nominations on particular nominees, turning the nominators into monogamous-like nominators? (Figure S1a, inset). Note that “polygamy” does not necessarily mean that the nominator gave up (lost motivation) in their commitment to a specific candidate and turned to another. It may also be the case of a nominator, whose candidates became Nobelists at their first or nearly first nominations, leaving the nominator with the possibility of committing to other candidates.

Figure S1 shows that a number of frequent nominators were often monogamous in their commitments, as they tended to advocate for a specific candidate until that candidate had been selected to receive the NPch. Once this goal was achieved, these frequent nominators shifted their focus to other nominees. A detailed description of the polygamous/monogamous character of nominators over the time period studied is provided in the Supporting Information (SI 1–5).

This triggers questions on the success of nominators. We turn to this subject in the next section.

#### 2.2.6 Nominator’s Success

We calculated the success (*s*) of a nominator as the fraction of a nominator’s commitments to nominees who eventually became Nobelists. Note that this is a presentist account to the success of a nominator, taking into account whether a nominee became a Nobelist *after* 1970. “Presentism” means the assessment of past commitments in terms of the outcome we know today of those commitments after 1970.^35^

Half of the nominators who submitted nominations between 1901 and 1970 were unsuccessful (*s* = 0) (Figure 5a). Only about a quarter of them were completely successful (*s* = 1). Intermediate successes (0 < *s* < 1) are more homogeneously spread over the population of nominators, with 10% of nominators having half successes (*s* = 0.5), about 4% achieving one or two-thirds of success (*s* = 0.33 and *s* = 0.66, respectively), and 1.4% attaining one and three-quarters of success (*s* = 0.25 and *s* = 0.75) (Figure 5a). The symmetry in the oscillating distribution of success values (Figure 5) is influenced by the mathematics of the fractions leading to the quantification of success, where most probable success values correspond to 0 and 1; 1/2; 1/3 and 2/3; 2/5 and 3/5; etc (SI 1–6, for the underlying combinatorics of success).

**Figure 5:**
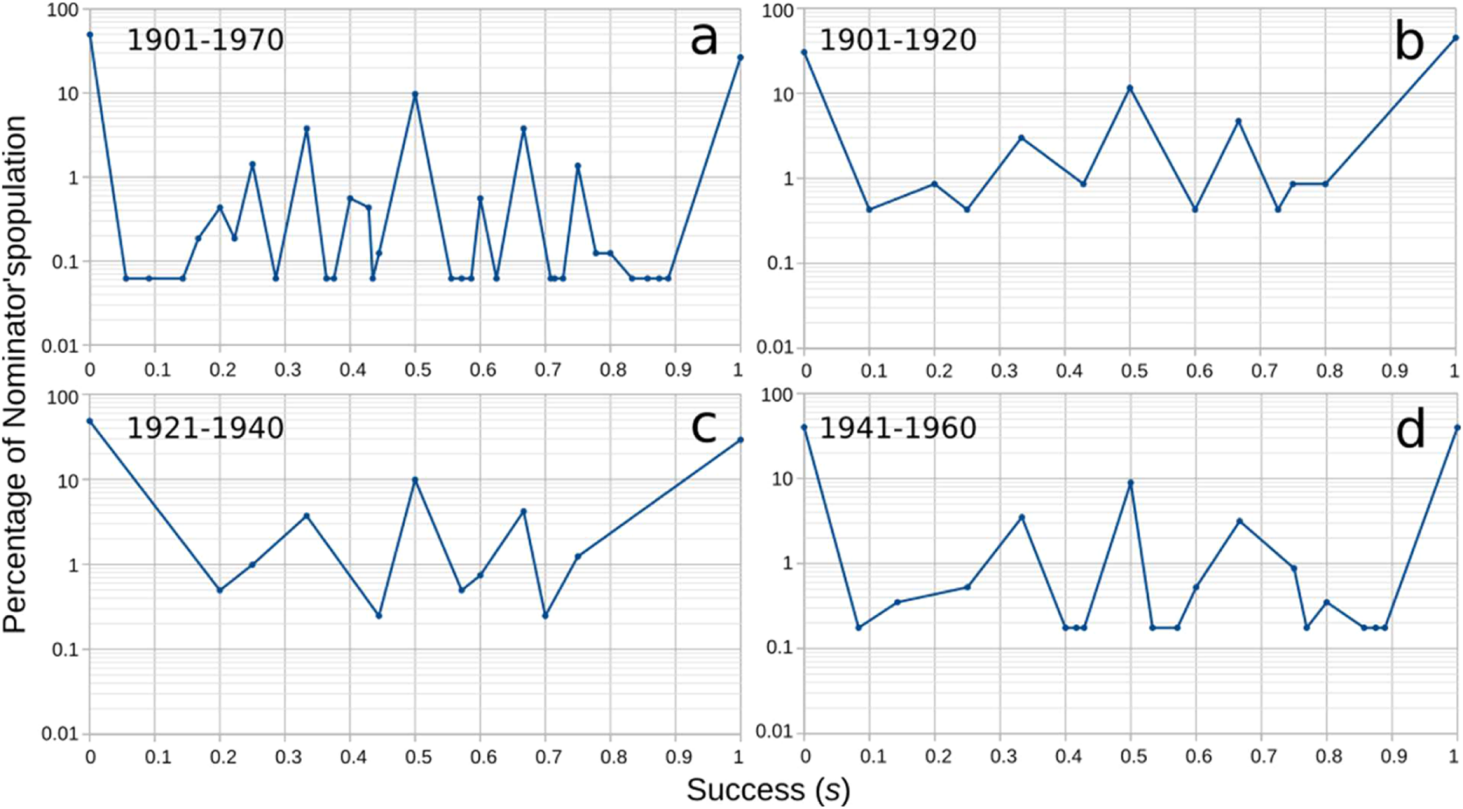
Nominator’s success. The success (*s)* of a nominator is defined by the number of commitments to nominees who eventually received the Nobel prize in chemistry, even after 1970, divided by the number of the nominator’s commitments. Plots in a–d depict the distribution of *s* values over the population of nominators in the four analyzed periods.

The probability of a success value for a given nominator is determined by the probability *p* that the Committee considers or not nominator’s nominations. This decision is shaped by various factors, including the nominators’ and nominees’ achievements and background. For example, their reputations, the “longevity” of nominee’s achievement, their discipline and, the alignment of the nominee’s discipline and achievement with Committee member’s professional biases, the disciplinary nature of previous NPch recipients, Committee’s personal biases, societal considerations, to name some of the biasing factors.^11,19,24,36–42^ The *p* results from the complex interaction of the just discussed factors. In fact, when nominations have *p* = 0.5 of being accepted by the Committee, success values are symmetrically distributed around *s* = 1/2 (Figure S2 (SI 1–6)). When the likelihood of acceptance is very low, say *p* = 0.1, most likely success values are concentrated in the region *s* < 0.5. In contrast, if nominations are highly accepted by the Committee, say *p* = 0.9, then success values are more frequent for *s* > 0.5.

In the first 20 years of the NPch, nominators’ commitments achieved more acceptance by the Committee than in later periods, as almost half of the nominators (45%) were completely successful, while 30% were unsuccessful (Figure 5b). This is presumably the result of the internationalistic spirit of those times and of the small size of the pool of nominators and nominees during those initial years of the NPch. The interwar period turned the initial trend upside-down as only 30% of nominators could send all their nominees to Stockholm, while about half of the nominators failed in their nominations (Figure 5c). This behavior was presumably caused by the post WWI scientific boycotts and antagonisms influenced by nationalistic stances,^5,34,43^ which led nominators to commit to fewer nominees (Figure 2b). Therefore, the likelihood that nominators’ candidates would have received the NPch dropped, increasing the number of unsuccessful nominators and reducing that of successful ones, as only a few nominators sent their candidates to Stockholm. The effect of the low number of commitments per nominator during the interwar period was also observed in the reduced set of values of success within the period (Figure 5c). As there were less commitments per nominator, the available fractions used for calculating the success also dropped. The period 1941–1960 witnessed an increased fraction of successful nominators such that the percentages of successful and unsuccessful nominators were approximately the same, spanning about 40% of nominators (Figure 5d). This is a consequence of the increase of commitments nominators had after WWII, which increased the likelihood of aligned nominations, therefore the likelihood of having more successful nominators.

The identities of the most successful nominators are shown in Figure 6. Several of them correspond to frequent nominators, who are also Committee members or members of the Royal Swedish Academy of Sciences (those in bold face in Figure 6). The Nobelist Ružička is an exception to this generalization (Figure 6a and d), as he was not part of the just mentioned institutions but, nevertheless, was a frequent nominator committing to 18 candidates within 55 nominations. Of these 18 commitments, 14 of them were to receive the NPch.

**Figure 6:**
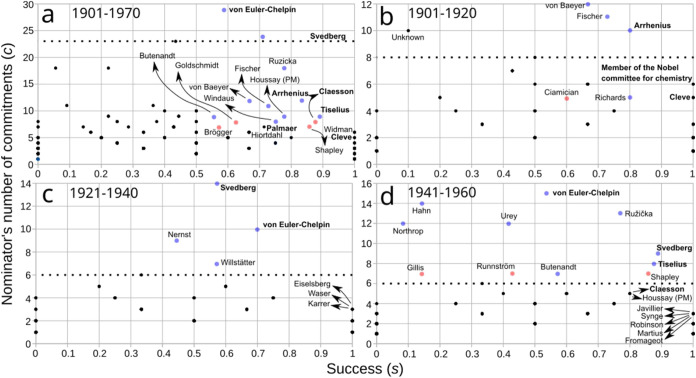
Distribution of nominators according to their success. Nominators are presented according to their success (*s*) calculated as the ratio between the number of the nominator’s commitments who become Nobelists and the total number of the nominator’s commitments. The identities of successful nominators (*s* > 0.5) are presented plus those of a few other frequent nominators. Black dots may correspond to more than one nominator. Blue dots indicate that the successful nominators are Nobelists (only those with more than four commitments are shown), while red dots indicate that the successful nominators are nonlaureates. Horizontal dotted lines separate regular from frequent nominators (Figure 3). Frequent nominators lie above the lines.

In the first two decades of the NPch, Arrhenius was a very successful frequent nominator (Figure 6b), as eight out of his 10 nominees received the NPch. Arrhenius’s influence was so strong that almost anyone who did not receive the NPch in the first two decades of the award failed to achieve Arrhenius’s support. Nobelists Emil H. Fischer and von Baeyer were also frequent, successful nominators in this period. Note how all six candidates by an unknown member(s) of the Nobel Committee for Chemistry as well as all five candidates of Committee member Per Theodor Cleve received the NPch, which turn these two Committee members as completely successful nominators.

The interwar period (Figure 6c) evidences the discussed small number of nominators and the lack of aligned commitments (Figure 1). Under these conditions, the likelihood of success was low. Note that completely successful nominators (*s* = 1) led at most three candidates to the NPch, while in the previous period, completely successful nominators led to six NPch. Here, again, Committee members were among the frequent successful nominators, which indicates that von Euler-Chelpin and Svedberg not only took over the nomination duties, but that they also persuaded the Committee and the Academy with their nominations. It appears that von Euler-Chelpin and Svedberg controlled the NPch in the interwar period, even though their commitments were not completely successful; their number of nominees who received the NPch surpassed the number of successes of the completely successful nominators (Figure 6c). Interestingly, Svedberg and von Euler-Chelpin became Nobel laureates themselves in this period (Figure 4). We note in passing that von Euler-Chelpin’s NPch was, as expected, motivated by his nominations, but unexpectedly by his self-nomination, which left him out of Committee’s work, leaving the burden of Committee’s activities on the four remaining members.^5^ This led Svedberg to rapidly wrap up a Nobel prize that could include Committee member von Euler-Chelpin.^5^ The list of frequent successful nominators in the first two decades of the NPch includes the Nobelist Willstätter, as four of his seven commitments received the NPch. Also in this interwar period, Anton von Eiselsberg, Ernst Waser, and Nobelist Paul Karrer were completely successful nominators, who committed each to three candidates.

The important role of Committee members as successful nominations did not decrease in the years to come, as evidenced in Figure 6d. For instance, Svedberg, Tiselius and von Euler-Chelpin committed to eight, seven, and seven, respectively, of the eventual Nobel laureates of that period.

Having discussed several aspects of the nomination process from the perspective of the nominators, now we turn to the nominee’s perspective.

### 2.3 Nominees

#### 2.3.1 Uneven Participation of Nominators Leads to an Uneven Pool of Nominees

In Section 2.2.2, we identified two distinct groups of nominators based on their level of participation: regular nominators and frequent nominators. This section explores how this uneven participation impacts the pool of nominees.

We analyzed the distribution of nominations and commitments over the population of nominees (Figure 7). In the period studied, there has always been a small fraction of nominees receiving many nominations and commitments. These nominees are located on the right of the distributions. In contrast, the vast majority of the nominees receive just a few nominations/commitments and they are observed on the left of the distributions. Hence, the uneven participation of nominators in the nomination process discussed in Section 2.2.2 leads to a pool of nominees with an uneven number of commitments and nominations.

**Figure 7:**
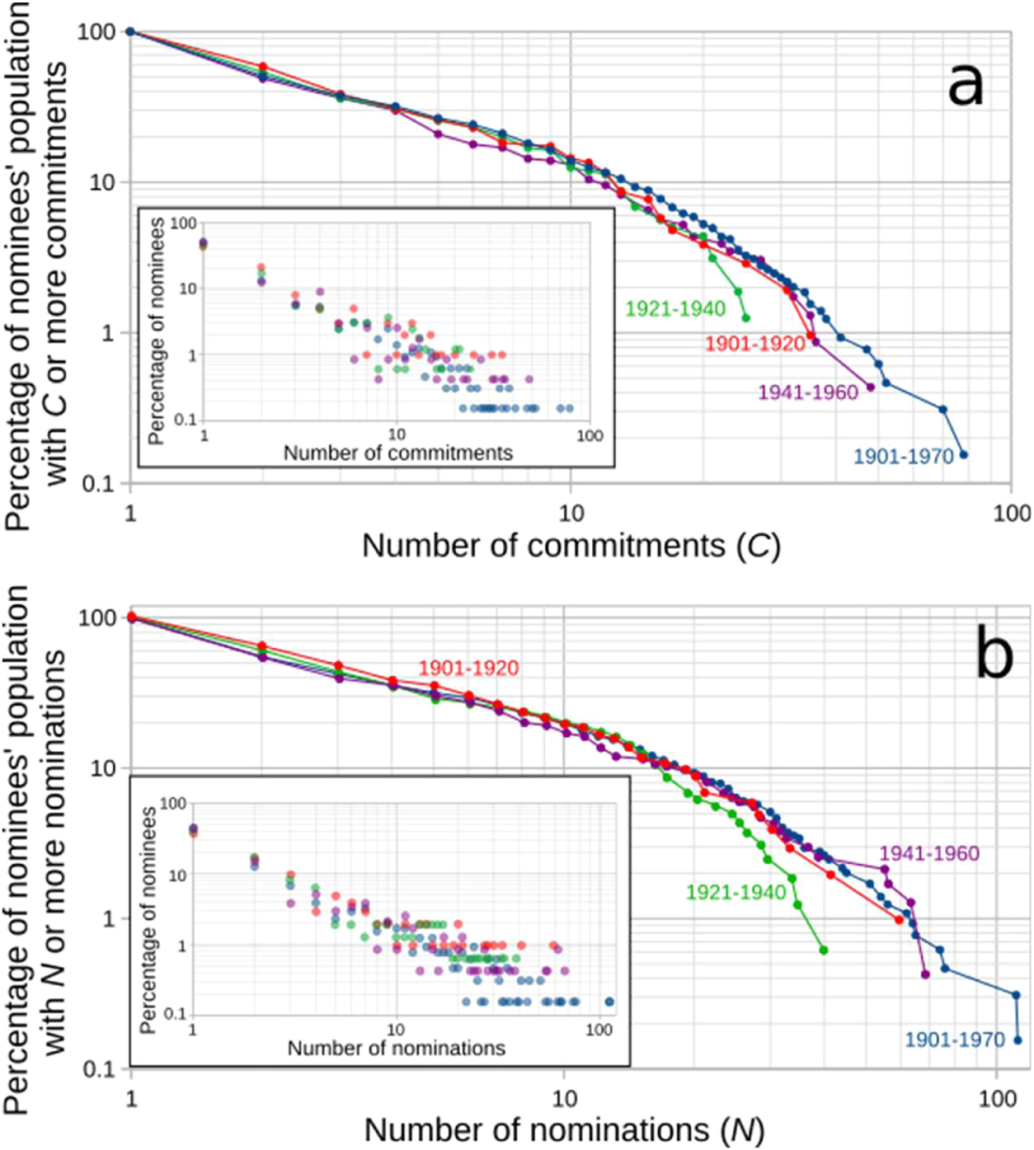
Nominees’ support. (a) Percentage of nominees receiving *C* or more commitments and (b) *N* or more nominations. Inset in (a): number of commitments per percentage of the nominees’ population. Inset in (b): number of nominations per percentage of the nominees’ population.

The distribution of commitments for the first two decades of the award (Figure 7a) indicates that about 1% of the nominees received 35 commitments, which is about 8% of the commitments that were experienced in those decades. These candidates constitute the pool of nominees frequently selected by nominators of those initial stages of the award. In terms of nominations (Figure 7b), the candidates receiving the largest number of nominations received 60 nominations (about 10% of the nominations submitted in this period).

The interwar period shows a shrinking of commitments in line with the shrinking number of nominations, especially evident for that fraction of nominees receiving more commitments than other nominees. Note, for instance, that about 1% of the nominees, instead of receiving more commitments than the 35 received in 1901–1920, actually received much less, in fact, 25 (Figure 7a). This shrinking of commitments is observed in the shift of Figure 7a (green) toward the left of the other plots for high values of the number of commitments. The same trend is observed for the number of nominations (Figure 7b, green). Standing on the side of the nominees, this is the result of the discussed perturbation of the nomination system, presumably caused by the lack of internationalization and by the boycotts and antagonisms emerging after WWI.^5,34,43^ As discussed in Section 2.3.1, this is also consistent with nominators being hesitant to commit to several candidates. It is very likely that the lack of an emerging group of candidates, strongly supported by commitments and nominations, posed a challenge for Committee members. This could have been taken as a justification by a few Committee members to intervene by stressing their roles as nominators and by fostering, in turn, their own research and disciplinary agendas.^5^ In the absence of highly supported nominees, the NPch was awarded only after a certain threshold of nominations or of commitments was reached. This hypothesis is examined in the following sections.

In the decades following the two WWs, the dynamics between nominators and nominees, reflected through their commitments and nominations, began to return to patterns similar to those seen before WWI. This shift is illustrated in Figure 7, where the purple data points align more closely with the trends depicted by the red data points. This was presumably motivated by aspects such as the reorganization and institutionalization of science after WWII and the rise of big science.^44,45^ Nominators increased their number of commitments as observed in 89% of the nominators lying in the region α in Figure 2a (purple), which is the same percentage of nominators in the same region between 1901 and 1920 (Figure 2a, red). Nevertheless, about 11% of nominators committed to seven or more nominees (region β in Figure 2a, purple) and 23% of nominators submitted more than 11 nominations (region β in Figure 2b, purple). The maximum number of commitments of a nominator within the period was 15 (by von Euler-Chelpin), who was the same nominator submitting the most nominations (37) (Figure 3). This produced a focused pool of possible awardees, where only 2% of the nominees were frequently nominated (more than 50 nominations (Figure 7b, purple)). For the sake of comparison with the previous periods, 1% of the nominees received 9% of the nominations submitted in this period.

The above results show the effect of the uneven participation of nominators upon the commitments and nominations received by nominees. Overall, the frequent participation of a small group of nominators led to a reduced set of strongly supported nominees. In fact, in each of the 20-year periods analyzed, 1% of the nominees gathered no more than 10% of the nominations submitted within each period. Note that it is in this small fraction of the population of nominees that the consensus of nominees discussed in Section 2.1 is particularly evident.

These results trigger several questions. To what extent did frequently nominated nominees correspond to Nobelists? We analyzed this question in the next section.

#### 2.3.2 Although Nobelists Received More Nominations than Non-Nobelists, Well-Supported Candidates Had no Secure Ticket to Stockholm

To analyze the effects of nominations and commitments upon the eventual selection of Nobel laureates, we calculated the correlation between the number of nominations and the number of commitments for nominees, which turned out to be very strong (0.968, *p*-value <2.2 × 10^–16^). Figure 8 indicates that the two groups of candidates, Nobelists and non-Nobelsits, have always been distinguished on their total number of nominations, though there are several exceptions. Nobelists have, in general, received more nominations than non-Nobelists, despite the fact that non-Nobelists will have more years during which they could be nominated. Thus, the number of nominations constitutes a proxy that differentiates Nobelists from non-Nobelists in a general statistical sense (SI 1–7). However, as seen in Figure 8, there are outliers to this trend, indicating cases of strongly nominated candidates for whom the number of nominations was not a decisive factor in leading them or not to Stockholm. There are also Nobelists who received very few nominations. In the former category, the leading example is Sir Christopher K. Ingold, who has been the most nominated candidate of the NPch, with 112 nominations by 78 nominators, but never received the award.^46^ Walter Reppe and Georges Urbain are also interesting cases of strongly supported candidates not receiving the award. In contrast, there was a chemist who received 111 nominations with 70 commitments who eventually did receive the NPch, that being R. B. Woodward in 1965.^46^ As seen in Figure 8a, their number of nominations rivals those received by Nobelists such as Nernst, Staudinger, and Linus C. Pauling. Indeed, several other well-supported nonlaureates received more nominations than those received by many Nobelists. This is observed by projecting the red dots of Figure 8a horizontally over the box-plot of Nobelists. This observation must be considered with caution, as several of the most deserving Nobelists received their Prize with one or just a few years of nominations, thus reducing the number of nominations they might have received had they not already received their NPch.

**Figure 8:**
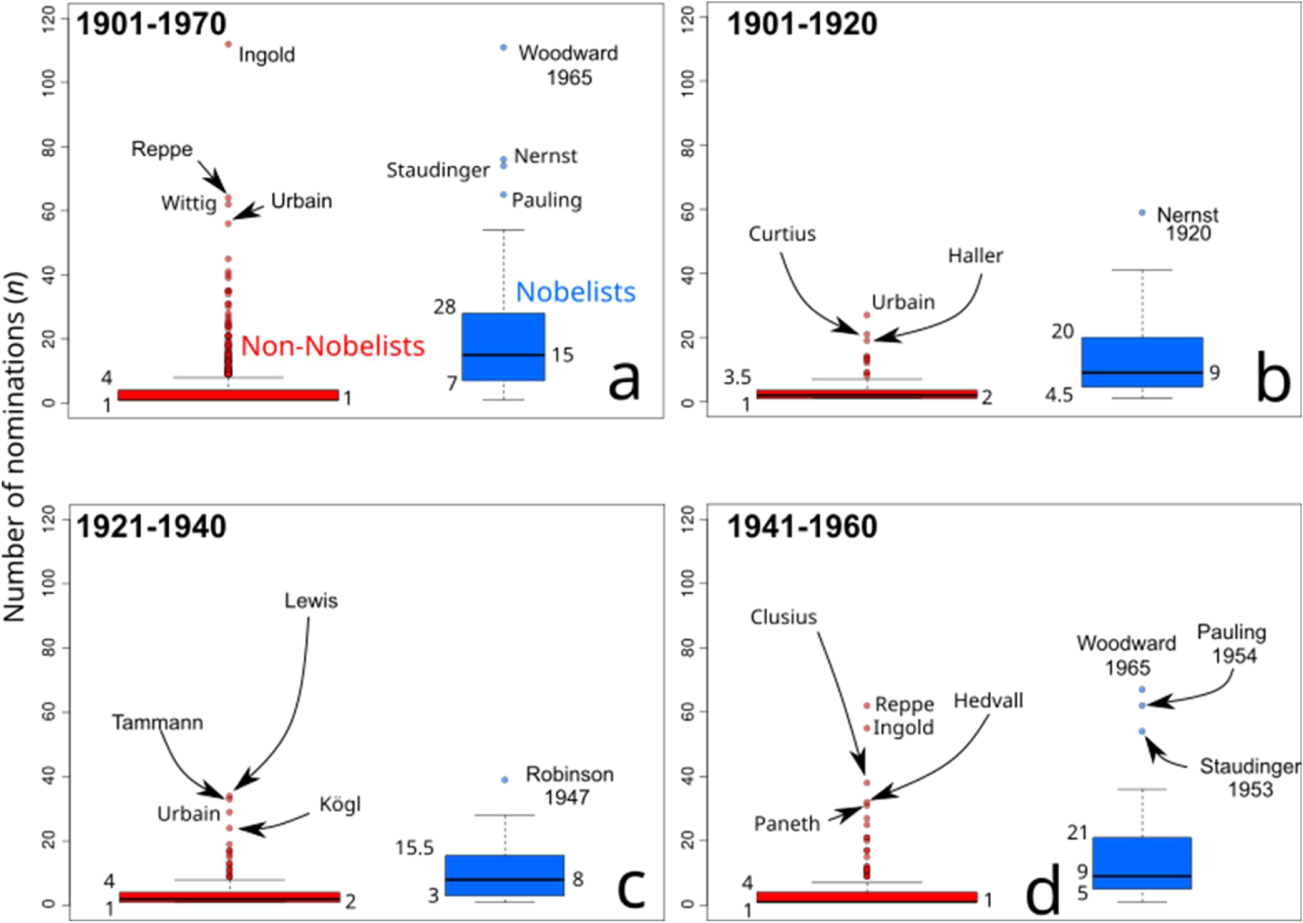
Distribution of nominations among Nobelists and non-Nobelists. The distribution of nominations for Nobelists and non-Nobelists is presented as box-plots, in blue for the former and in red for the latter. Boxes of these box-plots depict in their lower part the first quartile (Q1) of the data and in their upper part the third quartile (Q3), the distance between these quartiles corresponds to the interquartile range (IQR). The horizontal black line inside the boxes indicates the median (second quartile, Q2). Values of Q1, Q2, and Q3 are provided on the sides of the box-plots. Bottom whiskers correspond to Q1 – 1.5IQR and upper whiskers to Q3 + 1.5IQR. Outliers are indicated as colored dots, and the most extreme cases are labeled with the names of the nominees. In cases where the nominee is a Nobelist, the year of the award is provided.

In contrast, there are Nobelists who received few nominations by a few nominators. There are three extreme cases, namely Francis W. Aston and Arthur Harden, laureates in 1922 and 1929, respectively, as well as Urey, who received the award in 1934. Each of these chemists received the award with only one nomination.^46^

Throughout the 1901–1970 period, half of the non-Nobelists received no more than two nominations and three-quarters of non-Nobelists received no more than four nominations (Figure 8). In contrast, Nobelists have had a wider range of nomination records. One quarter of the Nobelists required no more than seven nominations. The maximum number of nominations of this small fraction of Nobelists has nevertheless oscillated over history. In the first two decades of the NPch, no more than five nominations were required for a quarter of the Nobelists. In the interwar period, as a consequence of the drop of the number of nominations (Figure 1a), 25% of the Nobelists were awarded with only three nominations, a number of nominations that increased between 1941 and 1960 to five (Figure 1a). In the decade between 1960 and 1970, half of those newly selected Nobelists received an unprecedented high number of nominations, namely up to 15 nominations (Figure 8a), which contrast with the up to nine of the previous history of the NPch (Figure 8b–d). This is likely related to the large number of nominations received after 1962 (Figure 1a). Between 1960 and 1970, three-quarters of the Nobelists received up to 28 nominations (Figure 8a), which is seven more nominations than those required by three-quarters of Nobelists between 1941 and 1960 (Figure 1d). This is also related to the surge in nominations received after 1962 (Figure 1a).

Thus, while it is true that Nobelists receive more nominations than nonlaureates, there is a mix of highly endorsed candidates that includes both laureates and nonlaureates. Among these well-supported individuals, the number of nominations introduces a degree of unpredictability, reflecting potential professional or personal biases in their journey toward the NPch. Within this group, the role of the Committee becomes crucial.

In the above discussion, we have focused on four time periods for nominations. But there is another type of time period worthy of investigation, that being the nomination time period, from the year of the nominees’ first nomination to the year of their last nomination. We analyzed this facet of the NPch in the next section.

#### 2.3.3 Nomination Periods

We next analyzed whether the distribution of years of nomination for Nobelists is different from that of nonlaureates (SI 1–8). Figure 9a indicates that the trend of Nobelists receiving more nominations than non-Nobelists is also true for nomination time. However, there is a certain degree of overlapping between the two groups of nominees, which can be observed by projecting the red box-plots horizontally over the blue box-plots in Figure 9. This overlap is not present at the level of the number of nominations received by the candidates (Figure 8).

**Figure 9:**
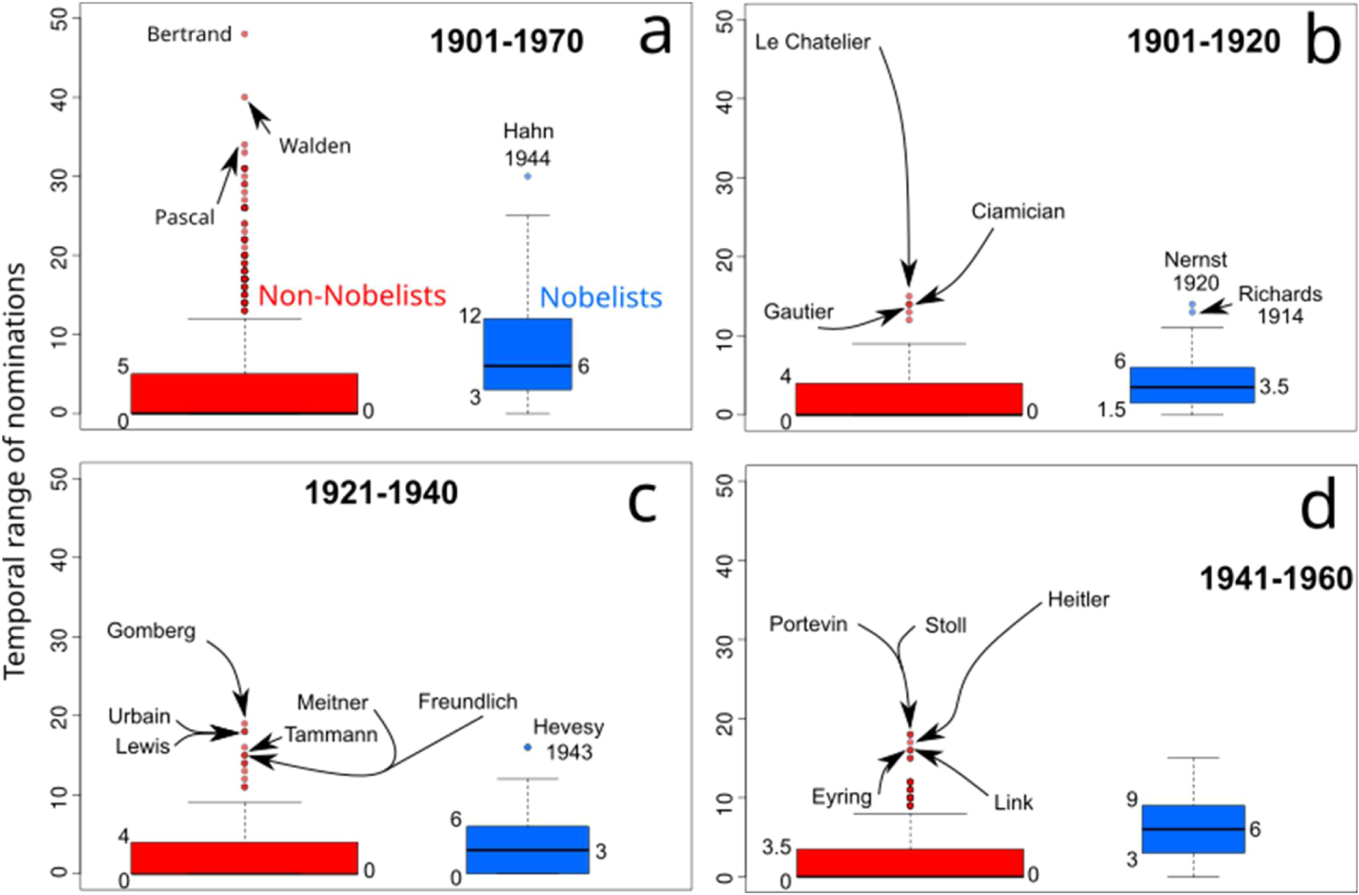
Distribution of years of nomination among Nobelists and non-Nobelists. The distribution of nomination years for Nobelists and non-Nobelists is presented as box-plots, in blue for the former and in red for the latter. The temporal range of nominations for each candidate was calculated as the difference between their last and first nomination year. Boxes of these box-plots depict in their lower part the first quartile (Q1) of the data and in their upper part the third quartile (Q3), the distance between these quartiles corresponds to the interquartile range (IQR). The horizontal black line inside the boxes indicates the median (second quartile, Q2). Values of Q1, Q2, and Q3 are provided on the sides of the box-plots. Bottom whiskers correspond to Q1 – 1.5IQR and upper whiskers to Q3 + 1.5IQR. Outliers are indicated as colored dots, and the most extreme cases are labeled with the names of the nominees. In cases where the nominee is a Nobelist, the year of the award is provided.

Figure 9a shows that Nobelists, overall, had a larger spread of nomination years than non-Nobelists. In all the periods analyzed, a quarter of Nobelists were nominated in no more than three different years, while half of the Nobelists went to Stockholm with no more than six years of nomination. By further extending the population to account for three-quarters of the Nobelists, we found that they received the NPch with up to 12 nomination years. In contrast, three-quarters of the non-Nobelists had more than five nomination years. Note that, as most of the non-Nobelists received a single nomination, Figure 9 shows that, over history, at least half of these nominees have been nominated in a range of zero years.

A comparison of the 20-year periods of this study (Figure 9b–d) with the entire analyzed period (Figure 9a) reveals that the last decade (1960–1970) witnessed a surge in the years of nominations that led to the NPch. This may be caused by the surge in the number of nominees observed after 1963 (Figure 1b), that is, more competition, which extended the waiting time, and likely the probability, to receive the NPch.

In the 1920s and 1930s (Figure 9c), Nobelists did not require many years of nomination to receive the NPch. This is presumably a consequence of the perturbation to the nomination system caused by the interwar period we have discussed before. It is in this period that the distribution of nomination years of Nobelists and non-Nobelists came closer to the analyzed history of the NPch. Note how in Figure 9c 75% of the non-Nobelists were nominated up to four years, while the same fraction of Nobelists only two further years. It is also in this period that 25% of the Nobelists went to Stockholm with a single nomination year.

Thus, although Nobelists and nonlaureates have had slightly different nomination time-periods, this variable is not a conclusive factor separating Nobelists from non-Nobelists, as there is a high degree of overlapping between the two groups of candidates.

A further aspect we wanted to explore was the interplay of nominations and their temporal span and its relationship with becoming or not a Nobelist. We explore such an interplay in the next section.

#### 2.3.4 Some Candidates Become Nobelists Accompanied by a Surge of Nominations

In this section, we address the question whether the sequence of annual nominations for all nominees (*nomination trajectories*) differentiates Nobelists from non-Nobelists and whether the community of nominators winds down its support for candidates who despite being often nominated, do not receive the NPch.

By analyzing the nomination trajectories for Nobelists and non-Nobelists, we found that the number of nominations is normally distributed for two-thirds of the laureates and for 87% of the nonlaureates (SI 1–9). The larger fraction of non-Nobelists having a normally distributed number of nominations is caused by the few nominations most of the non-Nobelists receive (Figure 8). These proportions indicate that 44% of the Nobelists deviate from a normal distribution of their nominations because they receive an atypical number of nominations, while only for 13% of the nonlaureates the same trend occurs. That is, 44% of the Nobelists have rather different nomination trajectories, whereas only 13% of non-Nobelists exhibit such distinctive trajectories. Some of these candidates with atypical nomination trends (trajectories) are depicted in Figure 10a,b.

**Figure 10:**
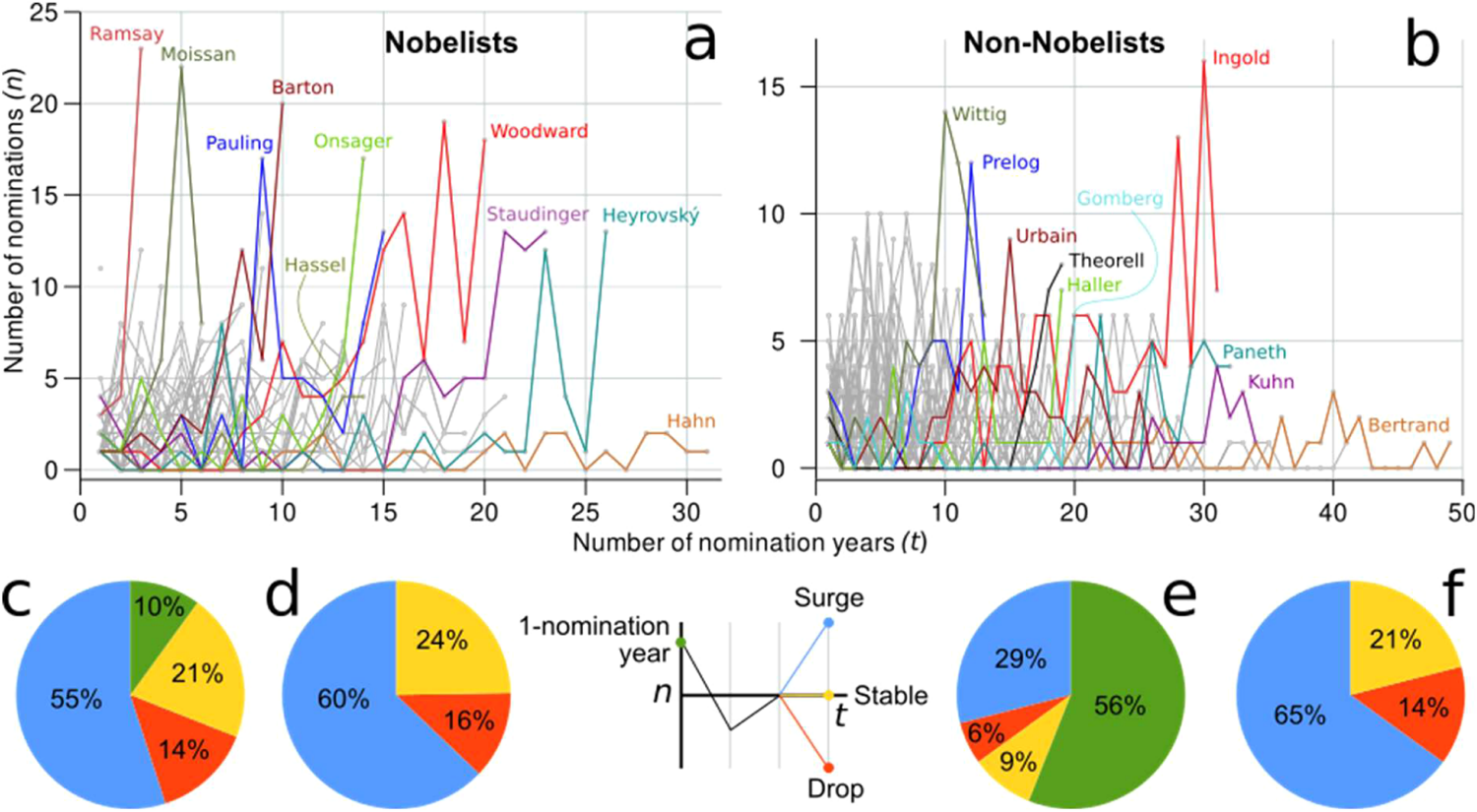
Nomination trajectories. Every nominee depicts a trajectory that is plotted as a curve indicating the annual number of nominations received from the initial nomination year until the final year of nomination within the period 1901–1970. For the bulk of candidates, their trajectories are colored gray. In (a), trajectories are presented for Nobelists, while in (b), for non-Nobelists. In both plots, some trajectories are highlighted (colored differently than gray), which are either relevant because they correspond to nominees departing from a normal distribution of their nominations or because they stand out as a consequence of some peaks in their trajectories. Panels (c) to (f) partition the population of nominees based on the number of nominations they received in their last two years of nomination. By considering the last two years of nominations, a candidate may have either a surge or a drop of nominations or even remain constant in the number of nominations. This is visually depicted in the scheme between panels (d) and (e). In (c), the partition is shown for Nobelists and in (d) also for Nobelists but by excluding nominees with only one year of nomination. In (e) the partition is shown for non-Nobelists and in (f) also for non-Nobelists but excluding nominees with only one year of nomination.

Note, for example, the surge of nominations for Henri Moissan the year before the award, or the case of Woodward with an increment of nominations about five years before receiving the Prize. Odd Hassel shows a surge about three years before his award that stabilizes in the last two years of nominations (Figure 10a). Among non-Nobelists with atypical nominations, we found famous nominees well deserving the prize (Figure 10b). Some of them have been discussed in the present document and a complete list is provided in SI 1–9. Figure 10b shows the surge of nominations for Ingold, especially in the last years of his nominations. A similar situation is observed for Gomberg, while Paneth had a peak of nominations a decade before his last nomination and reached the last nominations with a stable number of them.

We note that although nomination trajectories depend on the number of nominations and on the time span of those nominations, atypical nomination trajectories do not necessarily correspond to nominees that are outliers in terms of nominations (Figure 8) and of nomination periods (Figure 9). Thus, for instance, although Reppe and Nernst are outliers in Figures 8 and 9, they do not hold atypical nomination trajectories. In contrast, Nobelist Jaroslav Heyrovský depicts an atypical nomination trajectory (Figure 10) without being an outlier in Figures 8 and 9.

By considering the last two years of nominations, we found that 55% of laureates received more nominations in the awarding year than the previous year. This trend contrasts with the 14% laureates who received the NPch with fewer nominations than the previous year. Figure 10c shows that the percentage of laureates receiving the award with the same number of nominations as the year before is 21%, the remaining 10% of Nobelists achieved the award in the first year of nomination. Particular instances of Nobelists depicting these nomination trajectories are shown in Figure 10a. Hence, the proportion of laureates reaching the NPch year with a surge of nominations is about 4-fold that of laureates obtaining the prize with a drop of nominations. This provides quantitative support to the analyses of the minutes of the Committee meetings from 1901 up to 1950, which show surges in nominations for some Nobelists and indicate that some of those surges were motivated at the Committee level and that they were often the result of internal rivalries among Committee members or motivated to correct overseen nominees clearly deserving the prize.^5^ These observations are consistent with our recently published analyses of the last three years of nominations for a few Nobelists in chemistry,^46^ where we observed an up–down–up pattern for the analyzed laureates.

For the 562 nonrecipients of the NPch who were nominated from 1901 to 1970, their nomination trajectories lead to a distribution of candidates as shown in Figure 10e, where 56% of the candidates were nominated in but a single year, 29% reached the final nomination year with a surge of nominations, 9% with a stable figure and 6% with a drop of nominations. Some instances of non-Nobelists depicting these nomination trajectories are shown in Figure 10b. For an individual to be chosen when nominated in a single year is not like an athlete being chosen for the All-Star game based on their performance in that year. One can ask: How could it be that a person so deserving to be chosen with nominations in only one year was not nominated at all in any of the previous several years (other than in the first few years of the Nobel Prize)? One suggestion: an insider decision, that is, a decision by the Committee or possibly by a group of Academy members. This could be to ‘round-out’ an NPch for another individual who has received many nominations over several years, as discussed in our previous publication.^46^

The fractions of Nobelists and non-Nobelists with a surge, a drop, and a stable number of nominations in the last two nomination years become closer (Figure 10d and f). In fact, for about two-thirds of the candidates (Nobelists or not), their nomination trajectories finish with a surge of nominations, while about a quarter of the candidates receive the same number of nominations in the last two nomination years and about 15% of the candidates have a drop of nominations. That is, candidates with at least two nomination years cannot be distinguished by the final push of the community, which for both, Nobelists and non-Nobelists, is in about 63% of the cases of increased support, about 22% of steady support, while only for 15% of less support. It is against this background of supported nominees that the Nobel Committee decided in the analyzed period to whom to award the NPch. This nomination trajectory reflects the other side of the nomination coin: that community-wide pressure in support of certain nominees can be consequential. Certainly, the Committee and the Academy were not unaware of community sentiment, as their members are also members of the community! And the community of chemists is hardly a shy, reticent collection of hermits.

In summary, the nomination trajectories statistically do not distinguish Nobelists from non-Nobelists. Interestingly, about two-thirds of either Nobelists or non-Nobelists reach the last nomination year with a final increase in nominations. This indicates that nomination trajectories cannot be taken as a proxy to determine whether a candidate is a likely NPch recipient. Clearly, other aspects come into play in the decisions made by the Nobel Committee, especially personal and professional biases.

## 3 Conclusions

Our analyses revealed that there were three “organic” informal systems operating simultaneously that determined the recipients of the Nobel Prize for Chemistry (NPch):

- There were the meritorious recipients, certainly deserving of selection. This mode is heavily influenced by the power of community support, along with the achievements of the recipient.
- There was a system in which the professional and personal biases and prejudices of the Nobel Committee for Chemistry and perhaps also the Academy that was more influential than the nominations from the community, all of which extended beyond the realm of pure meritocracy.
- There were nominators and nominations that were outside both the meritocracy criteria and the Committee’s favorable if not controlling biases. In these occurrences, the nominators were known by the Committee not to be particularly influential; their nominations were not close to the meritocracy standards; and the nominees’ achievements did not fall within the realm of Nobel’s will, i.e., “to those who, during the preceding year, shall have conferred the greatest benefit on mankind.” These were situational awards.

We further conclude:

- There is no one-uniform nomination course or chronological trajectory that describes all successful (or unsuccessful) nominations. Some successful nominations are influenced primarily by community pressure, while others by the frequent nominations by a few Committee members and of Nobel Prize awardees. These individuals have had greater influence on the Committee than those of other nominators, and the alignment of candidates indicates a general consensus between Nobelists and Committee members.
- Of all the ex officio nominators, the most frequent were Committee members and a small group of Nobelists, not members of the Royal Swedish Academy of Sciences or professors of Scandinavian institutions of higher learning.
- There are nominations that are particularly influenced by the personal and professional biases of the members of the Nobel Committee. These influences go beyond the normal underlying perspectives that members of any award committee possess.
- As most nominations are for lesser-qualified candidates, typically one-off nominations, their fate is obvious, and their nominations are not pondered unduly.
- There have been two clearly differentiated groups of nominators: regular and frequent nominators. The former accounts for most of the nominators, who in their vast majority were nominated only once. Frequent nominators, in contrast, are a small group but were very focused on their own specific sets of nominations.
- The effect of the frequent participation of a few nominators caused the emergence of a reduced set of strongly supported nominees, not all of whom were eventually selected.
- While *in toto*, eventual Nobel laureates received more nominations over a longer period than nonrecipient, there was a mix of well-supported candidates, with some receiving the award and others never attaining it. In contrast, some Nobel laureates received very few nominations and received their Prizes within one or a very few years.
- There were more nominees than nominators, showing a degree of alignment among nominators on deserving candidates.

- Time periods within the 20th century have played a major role in the selection of the Nobel laureates, primarily because of nationalistic, sociological, and disciplinary biases.
- In the first 20 years of the NPch, nominators were more successful than in any other periods, likely as a consequence of the novelty of the award, where nominations and potential, extraordinarily highly meritorious nominees had not yet started to accumulate and even equilibrate.
- The participation of nominators was particularly disrupted by the World Wars. During these periods, the number of nominations decreased, and the Committees avoided awarding candidates proposed by frequent nominators due to a lack of consensus, presumably influenced by the nationalistic sentiments of the times. After WWII, the prewar nomination trends resumed, with an increase in nominators introducing a larger pool of nominees.
- The interwar period turned the initial nominators’ success upside-down, as only a few nominators secured the NPch for their candidates. The boycotts and antagonisms brought by WWI perturbed the dynamics of the nomination process, as suggested by others.^5,34^ It may well be that the Academy and the Committee saved the NPch from some level of quiescence by inserting its own actions.
- The 20 years after WWII showed an increase in the fraction of successful nominators. It actually reached a balance between the fraction of successful and unsuccessful nominators. This was caused by the increase of nominees after WWII, supported by a large percentage of aligned commitments that were received by the Committee.
- There were unanticipated powers executed by the Nobel Committee for Chemistry, perhaps even unanticipated by themselves. For instance, there may well be a natural social law leading to a participative imbalance in the nomination process, e.g., that frequent nominators dominate award programs. That is, there may be a variation of the Pareto law underlying the nomination process where a small fraction of nominators account for most of the successful nominations. If so, as our results show, one role of the Committee is to tune the variables of the Pareto law so as to avoid any handful of chemists, including members of the Committee themselves, from overly influencing the award system in chemistry.

We note our awareness that there are several important topics dealing with nominees that are amenable to large data analyses that we have either just barely touched upon (e.g., nationality bias^5,11,15,24^ ) or have not reported on (e.g., gender bias^13,24,47–50^ ). We hope to discuss these topics in the future.

## 4 Surveying the Status: Outlook

Chemistry has expanded rapidly over the recent decades. The NPch now incorporates the molecular aspects of the life sciences as much as it does pure chemistry and has the added the discipline of material science to its coverage as well.

Shepherding the Nobel Prizes is a special honor for the Academy and its Committee, but that honor comes with a weighty burden. The members are mostly active academics in their own right. Consider the increasing workload as a function of the number of nominations. In pre-WWII times, there was an average of ca. 37 nominations each year. In the 1960s, there were 143 annual nominations yearly. In 1970, there were 159 nominations. How many were there in 2024? Two hundred, 300, even more, and in more subdisciplines of chemistry?

The Committee for Chemistry handles this significant workload associated with this increased scientific scope while maintaining the same number of Committee members as during the inception of the award at the beginning of the 20th century. The Committee now relies on Adjoint members and confidential consultants chosen at will. But nonetheless, the Committee is still composed of a small group of (primarily) Swedish chemists and life scientists with the occasional Swedish physicist or material scientist—all of whom have their own professional responsibilities. And what they do is so highly visible.

We highlight the reality that the Committee members are the hosts, the invitees (for nominations), the administrators (they select and invite various confidential consultants), the judge, the jury, and the discussants who provide their recommendation to the Royal Swedish Academy of Science. The Academy also has the influence, if not the power, to make changes and the ability to make recommendations to the Nobel Foundation regarding possible changes to the Nobel Prize system.

In the most recent decades, the system does seem to work well and with intent. Above a somewhat quiet background of noise, there seem to be far fewer individuals of the highest caliber who really ought to have received the Nobel Prize in their lifetimes, yet did not. We have our own favorites and disappointments,^18,22,51,52^ as do our colleagues,^9,53–55^ and we are sad for them^51^ and for the Nobel Prize in Chemistry, which would have been honored to have all of our favorites among the chosen.

What will become of the Nobel Prize in the future? One of this journal’s peer reviewers asked, “In the modern age, the world is much larger and so is science. Will the Nobel Prizes become an anachronism, much like the titles conferred by British monarchs of the past?” We think, hope, and indeed expect that the Nobel Prizes will continue as focal points of honor, acknowledgment, and celebration of the best of human achievement, to both the relevant scientific communities and to the world at large.

## Supplementary Material



## Notes

Data treatment. Link to Code, Treatment of outliers, Section of “Active Nomination Time of Nominations,” Section on “Mathematics of the Success Function,” Section on “Statistical Tests Supporting the Separation of Nobelists from Non-Nobelists in Terms of Their Number of nominations,” Section on “Normality Tests for Nomination Trajectories,” All sections contain appropriate references (PDF)

## Notes

Dedicated to the memory of Robert H. Grubbs (1942–2021), a well-deserving Nobel laureate (2005).
