# Supplementary material for: How the Nobel Committee for Chemistry Has Shaped the Nobel Prize: Historical Trends Based on the Nobel Prize Nomination Archive
Source: ACS Omega. 2025 May 13;10(20):20078–94. doi: 10.1021/acsomega.4c08461 (PMC12120657; doi:10.1021/acsomega.4c08461)
Supplement: Supplementary file 1 [file ao4c08461_si_001.pdf]

How the Nobel Committee for Chemistry Has Shaped the Nobel Prize: Historical Trends Based on the Nobel Prize Nomination Archive

Jeffrey I. Seeman[1,*], Juan Amaya[2], and Guillermo Restrepo[3,4,5,*]

1 Department of Chemistry, University of Richmond, Richmond, Virginia, 23173 USA
2 Technische Universität Dresden, 01062 Dresden, Germany
3 Max Planck Institute for Mathematics in the Sciences, Inselstraße 22, 04103, Leipzig, Germany
4 Interdisciplinary Center for Bioinformatics, Leipzig University, Härtelstraße 16-18, 04107 Leipzig, Germany
5 School of Applied Sciences and Engineering, EAFIT University, Medellín, Colombia

Supporting Information

SI 1-1 Data

We dumped the Nobel prize nomination archive on September the 5th 2022 (code available in SI 6.2), which contains information on the nominations associated with the prize between 1901 and 1970. As our interest is the Nobel prize in chemistry (NPch), we considered only all nominations associated with this prize. This led us to analyse the nominator-nominee relationships among 1,991 scientists, which amounts to 4,325 nominations distributed in 3,159 commitments of the sort nominator → nominee (master file available in https://github.com/cshjsc/prizes_history as master.csv).

SI 1-2 Code

Code to dump information from the Nobel prize nomination archive is available in https://github.com/cshjsc/prizes_history, which also contains all files used in this research.

SI 1-3 Removing outliers of Figure 2 and fittings for region β

Turning points in plots depicted in Figures 2a and 2b were detected based on the minimal value of percentage of nominators attained in each plot. Points on the right of the turning point were fitted using a linear regression $P_{\beta}(X) \sim X^{m}$ (or equivalently $log\left(P_{\beta}(X)\right) \sim mlog(X)$), with *m* being the slope of the fitting line. In Figure 2a, the fitting procedure required omitting a few points of the plots for 1901-1920 and for 1941-1960. The omitted points correspond to those deviating from the linear trend observed in Figure 2a. Likewise, in Figure 2b a few points were omitted following the same procedure. Values of *m* and γ reported in the insets of Figures 2a and b correspond to those resulting from the fitting procedure here described.

SI 1-4 Active nomination time of nominators

To calculate the active time of Academy members as nominators, we took a random sample of the more than 2,900 members that have belonged to the institution and that were active in the analyzed period 1901-1970. The information was retrieved from https://en.wikipedia.org/wiki/Category:Members_of_the_Royal_Swedish_Academy_of_Sciences?from=Dj. The size of the sample was of $\sqrt{2900} + 1 = 55$ Academy members. The active time of each member was calculated as the difference between their year of death and their year of membership to the Academy. In cases where the academician was appointed to the institution before the instauration of the NPch, we took 1900 as the initial year of potential activity as nominator. A table containing this information is shown in the file random_academy.csv, which can be found at https://github.com/cshjsc/prizes_history. This information leads to an average potential nomination time of 25.4 ± 2.0 years. The actual nomination time of Academy members could not be calculated, as only six out of the 55 randomly chosen Academicians ever made nominations during the studied time frame.

The active nomination time of Nobelists was calculated in a similar manner. For each Nobelist the difference between the year of death and the year of the award was

calculated. Based on those differences the mean value of active time as nominators for Nobelists was calculated. A table containing the year of death, the awarding year and the difference is shown in the file Nobelists-active-time.csv, which can be found at https://github.com/cshjsc/prizes_history. This information leads to an average possible nomination time of 23.8 ± 1.4 years. The actual nomination time of Nobelists was calculated using the difference between the last and the first nomination years of each Nobelist; this leads to 15.0 ± 1.4 years.

For Committee members, their time of service as Committee members was calculated as the difference between their last year of service and the year of appointment as Committee member. This leads to an average time of service of 20.2 ± 2.1 years. The actual nomination time of Committee members was calculated by computing the difference between the last and the first nomination years of each Committee member; this leads to 21.7 ± 2.9 years.

The active time of nomination of ad-hoc nominators was calculated based on the difference between the year of the last and the first nomination submitted by non-Nobelist and non-Committee-member nominators. This produced an average nomination time of 3.59 ± 0.17 years. The information for this calculation was extracted from the master document discussed in section SI1-1. A file with the list of resulting nominators is found in the file nominators-noNobelists-noCommittee.csv, which can be found at https://github.com/cshjsc/prizes_history.

SI 1-5 Monogamous and polygamous nominators

Figure S1a shows the spread of Karl J. Freudenberg's nominations (the nominator with the largest superabundance of nominations (41) (Figure 3)), which reveals that he was very committed to a single nominee, namely to Hans L. Meerwein, with 13 nominations, which actually constitutes the commitment with the highest number of nominations within the disclosed archives. Nevertheless, Meerwein never received the NPch. Out of Freudenberg's 11 commitments, four received the NPch.

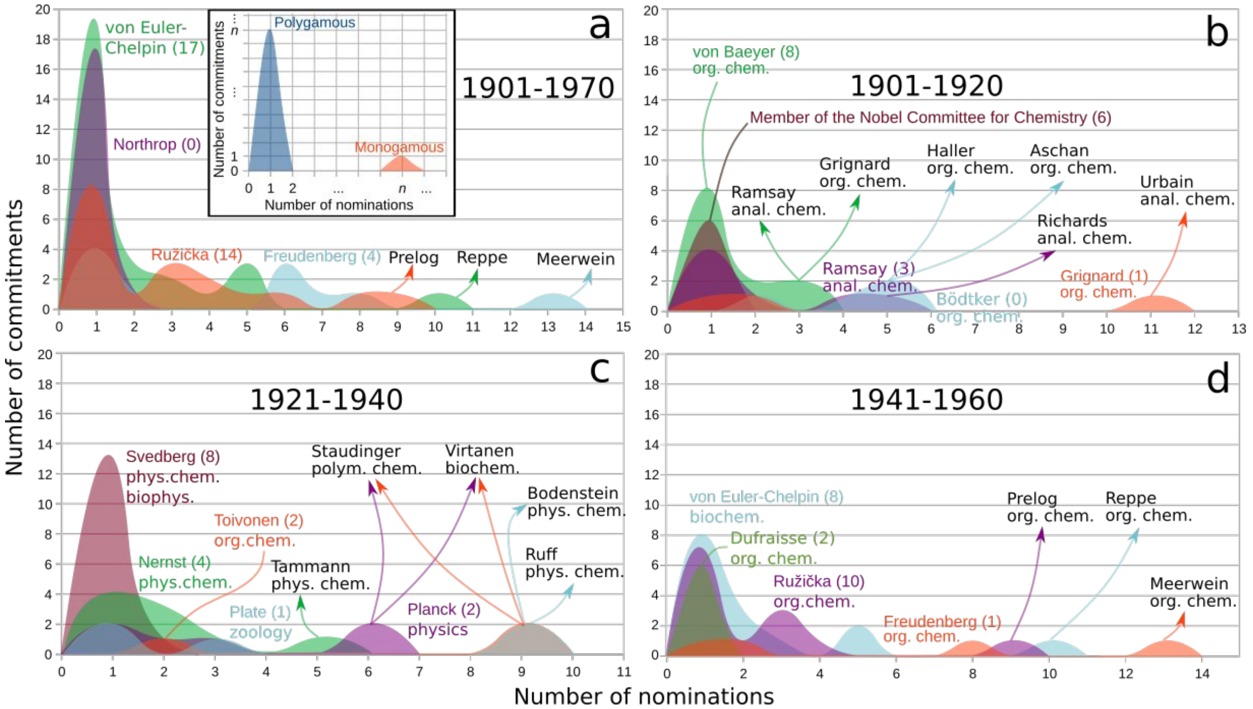

Figure S1. Distribution of nominations for a few selected nominators, including frequent nominators. Each nominator's distribution and the name of the nominator are equally colored. For each nominator the number of nominees who eventually became Nobelists is provided in parenthesis. Right-most part of each distribution houses the most supported nominee(s) for each nominator, whose name(s) are provided in black as emerging arrows from nominator's distributions. Nominators and nominees are also labeled by their field of expertise. Inset in (a): Nominator's extreme cases, from polygamous nominators (submitting one nomination for *n* nominees) to monogamous nominators (submitting *n* nominations for a single nominee). In (b) the identities of the "Member of the Nobel Committee for Chemistry" have not been yet disclosed by the Nobel Foundation.

Another nominator with a superabundance of nominations was Ružička, who committed to 18 nominees with a superabundance of 37 nominations (Figure 3a). Ružička spread these nominations rather homogeneously (Figure S1a). He was among the most successful nominators from a percentage perspective (section 2.2.6). His most nominated candidate was his former PhD student, ETH Zürich colleague, and fellow Croatian, Vladimir Prelog, who received the NPch in 1975. Von Euler-Chelpin, a member of the Academy and of the Committee for 17 years and also a Nobelist (Figure 4a), was another nominator with a superabundance of nominations. He distributed his 31 superabundant nominations rather homogeneously over his 29 nominees (Figure 3a). Of von Euler-Chelpin's candidates, Walter Reppe was the nominee attaining most nominations (10 nominations). That is, out of 31 von Euler-Chelpin's superabundant nominations, Reppe received nine of them. As shown in Figure S1a, the other superabundant nominations from von Euler-Chelpin were distributed as follows: three candidates received four nominations each, one nominee received three, two candidates two nominations each and three nominees one nomination each. Of these, four became Nobelists. For the sake of comparison, we included in Figure S1 several cases of polygamous nominators, who invested a few nominations for each of their numerous commitments. These nominators lie close to the diagonal in Figure 3. A relevant one-shot yet polygamous nominator was John H. Northrop, with 18 commitments

through 19 nominations. He submitted two nominations for Alfred Gierer (Figure S1a). Nevertheless, none of Northrop's nominees received the NPch.

In the initial years of the NPch, Eyvind Bödtker, Victor Grignard, Sir William Ramsay and von Baeyer had the largest superabundance of nominations, while an unspecified (by the Nobel nomination information) nominator, a member of the Nobel Committee for Chemistry (which could actually be several Committee members) was a polygamous nominator committing to several nominees (Figure 3b). Bödtker concentrated his nominations on two nominees: Albin Haller and Ossian Aschan (Figure S1b), while von Baeyer spread single nominations on eight nominees and only advocated three times for Ramsay and Grignard (Figure S1b). As well as von Baeyer, Ramsay nominated four nominees with a single nomination and advanced five nominations for Theodore W. Richards (Figure S1b). Grignard, in turn, is the most monogamous nominee in this period. Although he nominated Haller twice and Willstätter once, Grignard decidedly nominated Georges Urbain 11 times. We discuss the reasons underlying this particular support for Urbain below.

In the interwar period, Ludwig Plate, Niilo J. Toivonen, Planck and Nernst were nominators with a superabundance of nominations, while Svedberg and Julius Wagner-Jauregg submitted just about one nomination per nominee (Figure 3c). Plate, a zoologist, submitted nine fruitless nominations for Otto Ruff and the same amount for Max Bodenstein. Likewise, Toivonen pushed for Hermann Staudinger and Artturi J. Virtanen with nine nominations each. These two nominees received the same number of nominations from Planck, who, nevertheless, distributed his superabundance of 13 nominations homogeneously over his nominees (Figure S1c). Nernst spread homogeneously his nominations, only advocating with five nominations for Gustav Tammann. Toivonen, in turn, decidedly supported Staudinger and Virtanen with nine nominations each. Figure S1c presents the less monogamous case of Svedberg, who committed to 14 nominees and only in one case he nominated twice, namely to Nernst (Figures 3c and S1c). In the years to come, Svedberg never submitted more than two nominations per nominee. We will see in section 2.2.6 that Svedberg's polygamous character is a consequence of his success as nominator, the very best reason to be a polygamous nominator!

The 1941-1960 period depicts von Euler-Chelpin, Freudenberg and Ružička as the nominators with the largest superabundance of nominations, while Northrop, Jan Gillis and Charles Dufraisse behaved as one-shot nominators (Figure 3d). It was in this period that von Euler-Chelpin strongly advocated for Reppe (Figure S1d) and that Freudenberg submitted 13 unsuccessful nominations for Meerwein. Likewise, the kind of homogeneous distribution of nominations of Ružička is evident in this period (Figure S1d).

As explained by disciplinary bias, many nominators' most supported nominee belongs to the nominator's research subdiscipline, as we exemplified in Figure S1 for a select group of nominators, including frequent nominators. There are, however, some exceptions as is the case of von Euler-Chelpin (biochemistry) for Reppe (organic chemistry); Grignard (organic chemistry) for Urbain (analytical chemistry); and Plate (zoology) for Ruff (physical chemistry) and Bodenstein (physical chemistry). The commitment of von Euler-Chelpin to Reppe may have been motivated as a counter to the WWI bias against nominations to German chemists.[1–3] Grignard's commitment to Urbain is presumably related to the post-WWI priority dispute on the discovery of Hafnium, in which Urbain was involved.[4] This is an

instance of nationalistic support, where the famous French chemist backed his compatriot. The role of nationalism in the Nobel Prize (in all fields) has been recently studied.[5] Plate's commitments to Ruff and Bodenstein are puzzling, because there is no clear connection between nominator and nominees, besides all three of them being Germans. Interestingly, the zoologist Plate not only nominated Ruff and Bodenstein several times but also other chemists such as Heinrich O. Wieland (Nobelist in 1927) and was also an active nominator in physics. This active role as nominator was possible by his membership to the Royal Swedish Academy of Sciences.

SI 1-6 Mathematics of the success function

The success of a nominator is quantified as *s* = *W*/*c*, where *W* stands for the number of commitments of the nominator such that the nominee eventually received the Nobel Prize and *c* corresponds to the total number of commitments the nominator has. Given that *W* and *c* take only positive integer figures, *s* is a reduced fraction between 0 and 1 (which we express as percentages in the main text). Hence, the range of values of *s* is not the continuous interval $[0,1] \in R$ but a discrete sample of fractions between 0 and 1 ($[0,1] \in Q^{+}$). Table SI-1 shows the possible values *s* may take for particular *c* values. We collect those *s* figures in the sequence F_c, which arranges them in non decreasing order. Possible realizations of *c* commitments are given by sequences of Ws and Ls, that is, of commitments leading to the Nobel Prize (win) and of commitments not leading to the Prize (lose). Note that F_c sequences are a particular subset of Farey sequences.[6]

Table SI-1. Instances of the number of commitments *c* of a nominator and their possible realizations involving successful (W) and not successful commitments (L). F_c gathers the possible success values for *c* commitments.

C	Possible outcomes	Fc
**1**	L, W	(0,1)
**2**	LL, WL, LW, WW	(0,½,1)
**3**	LLL, WLL, LWL, LLW, WWL, LWW, WLW, WWW	(0,⅓,⅔,1)

Thus, if the nominator commits to *c* nominees, the probability *P*(*W*) of obtaining *W* successes is given by the binomial distribution:

$$P(W) = \binom{c}{W} p^{W}(1-p)^{c-W}$$

Where $p \in [0,1] \in R$ corresponds to the probability of obtaining *W* in a given commitment. That is, *p* measures the probability that nominator's commitment be taken by the Nobel Committee. If the probability that the Nobel Committee follows nominator's suggestion is ½ (that is, if it is equally likely to be heard of not by the Nobel Committee), the expected value of $P(W)$ is *c*/2. This follows from the expected value of the binomial distribution $E(W) = cp$. A nominator whose commitments are always heard by the Nobel Committee has *p* = 1, so

the expected value of their success is 1. In contrast, a nominator never heard has an expected value of success of 0.

If we now consider the whole collection of nominators, who have different number of commitments, then we need to consider the probability of finding each success value *W*/*c* over the whole collection of possible commitments. For this, let us assume that the maximum number of commitments of the sample of nominators under consideration is *c*'. Then, we need to find the probability of finding *W*/*c* in all possible simple fractions allowed by *c*'. This boils down to determine the number of sequences F_c containing *W*/*c*, which for *c*' corresponds to those *c* dividing *c*'. Thus, the probability of finding *W*/*c* in the possible outcomes resulting from up to *c*' commitments is

$$P(s = W/c)_{c'} = \frac{\sum \binom{y}{y/c} p^{y/c}(1-p)^{y-y/c}}{\sum\sum \binom{c}{W} p^{W}(1-p)^{c-W}}$$

With *y* being a *c* dividing *c*'. Hence, if the maximum number of commitments of a nominator is *c*' = 10, then, for instance the probability of observing *s* = ½ is 0.17 (with *p* = 0.5) while that of *s* = 0 or *s* = 1 is 0.099. In Figure S2 we show the behavior of *P*(*s* = *W*/*c*) for different values of *p*. It is observed how even neighboring success values have different probabilities of occurrence and how the shape of the distribution is affected by *p*. For instance, for an scenario where commitments are equally likely of being accepted by the Nobel Committee (*p* = 0.5), it is clear that only about 17% of the commitments will be taken by the Nobel Committee. An scenario where most of the commitments are disregarded by the Nobel Committee (say *p* = 0) turns the most popular success value *s* = 0, with about 60% of the commitments being turned down. The same percentage is observed for the scenario where 90% of the commitments received by the Nobel Committee are taken into account (say *p* = 0.9).

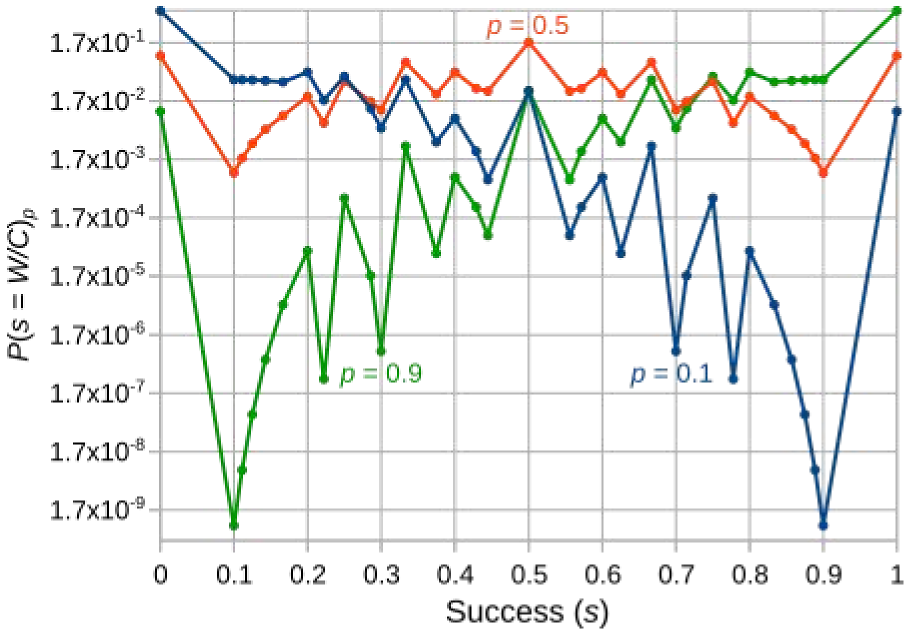

Figure S2. The hypothetical scenario of 10 commitments with its associated *s* values weighted by the probability *p* that the Nobel Committee accepts the commitment. Note how low *p* values turn more probable low *s* values, while high *p* figures the converse.

SI 1-7 Statistical tests supporting the separation of Nobelists from non-Nobelists in terms of their number of nominations

To analyze whether Nobelists are separated from non-Nobelists, we conducted Welch Two Sample t-tests and Mann-Whitney U tests over the nominees corresponding to each of the temporal periods studied, namely 1901-1970, 1901-1920, 1921-1940 and 1941-1960. Each nominee was characterized by their number of nominations within the period studied and by a label indicating if the candidate was a Nobelist or not. The files used to compute these statistics are located in https://github.com/cshjsc/prizes_history and correspond to the following documents, for each respective period: Nnominations-Nnominators-WorL.csv, Nnominations-Nnominators-WorL-1.csv, Nnominations-Nnominators-WorL-2.csv and Nnominations-Nnominators-WorL-3.csv. Table SI-2 gathers together the statistics of these tests.

Table SI-2. Summary of statistics of the Welch Two Sample t-test and Mann-Whitney U test run over data on number of nominations for the set of nominees.

	**Welch Two Sample t-test**			**Mann-Whitney U tests**	
**Period**	t	Degrees of freedom	*p*-value	W	*p*-value
**1901-1970**	-7.0132	82.714	,5.84×10^{-10}	5852.5	$< 2.2 \times 10^{-16}$
**1901-1920**	-3.554	24.642	0.001566	317	3.449×10^{-7}
**1921-1940**	-3.8663	44.178	0.000359	1115.5	2.271×10^{-6}
**1941-1960**	-4.0193	40.643	0.0002462	1319.5	6.795×10^{-11}

SI 1-8 Statistical tests supporting the separation of Nobelists from non-Nobelists in terms of their nomination times

To analyze whether Nobelists are separated from non-Nobelists, we conducted Welch Two Sample t-tests and Mann-Whitney U tests over the nominees corresponding to each of the temporal periods studied. Each nominee was characterized by their number of years of nomination within the analyzed period and by a label indicating if the candidate was a Nobelist or not. The files used to compute these statistics are located in https://github.com/cshjsc/prizes_history and correspond to the following documents, for each respective period: 1901-1970.csv, 1901-1920.csv, 1921-1940.csv and 1941-1960.csv. Table SI-3 gathers together the statistics of these tests.

Table SI-3. Summary of statistics of the Welch Two Sample t-test and Mann-Whitney U test run over data on nomination times for the set of nominees.

	**Welch Two Sample t-test**			**Mann-Whitney U tests**	
**Period**	t	Degrees of freedom	*p*-value	W	*p*-value
**1901-1970**	4.9035	118.22	3.032	12394	3.495×10^{-14}
**1901-1920**	1.9018	37.722	0.06486	621	0.005734
**1921-1940**	1.5456	60.86	0.1274	1753.5	0.009985

**1941-1960**	4.8927	55.859	8.825	1787	3.737×10^{-8}

SI 1-9 Normality tests for nomination trajectories

We collected the nomination trajectories for each Nobelist. For the 55 Nobelists having more than two nomination years we ran the Shapiro–Wilk normality test. The condition of minimum two nomination years results from the minimal set of data required to run the test. The statistic and *p*-value for each of the 55 Nobelists is found in https://github.com/cshjsc/prizes_history in the file shapirowilkNPres.csv. If the *p*-value is lower than 0.05 those Nobelists have no normal distribution of nominations.

The same procedure was applied to each of the 103 non-laureates with more than two nomination years. The results are found in https://github.com/cshjsc/prizes_history in the file shapirowilkNWres.csv.

The code for analyzing the nomination trajectories of the last two years of non Nobelists having two or more nomination years is found in https://github.com/cshjsc/prizes_history as trajectories_last_years.txt.

References

(1) Friedman, R. M. *The Politics of Excellence: Behind the Nobel Prize in Science*; Times Books, 2001.
(2) Crawford, E. Internationalism in Science as a Casualty of the First World War: Relations between German and Allied Scientists as Reflected in Nominations for the Nobel Prizes in Physics and Chemistry. *Soc. Sci. Inf.* **1988**, *27* (2), 163–201. https://doi.org/10.1177/053901888027002001.
(3) Reinbothe, R. [The boycott against German scientists and the German language after World War I]. *Dtsch. Med. Wochenschr. 1946* **2013**, *138* (51–52), 2685–2690. https://doi.org/10.1055/s-0033-1359937.
(4) Scerri, E. *A Tale of Seven Elements*; Oxford University Press USA, 2013.
(5) Gallotti, R.; De Domenico, M. Effects of Homophily and Academic Reputation in the Nomination and Selection of Nobel Laureates. *Sci. Rep.* **2019**, *9* (1), 17304. https://doi.org/10.1038/s41598-019-53657-6.
(6) Niven, I.; Zuckerman, H. S. *An Introduction to the Theory of Numbers*; Wiley, 1972.
